# Comparative analysis of malignant pleural effusion and peripheral blood reveals unique T cell signatures associated with survival in mesothelioma patients

**DOI:** 10.1093/oxfimm/iqaf008

**Published:** 2025-12-24

**Authors:** Nicola Principe, Kofi L P Stevens, Amber-Lee Phung, Melanie McCoy, Joel Kidman, Ali Ismail, Alistair M Cook, Abha Chopra, Mark Watson, Bruce W Robinson, Jenette Creaney, Y C Gary Lee, Jason Waithman, W Joost Lesterhuis, Richard A Lake, Anna K Nowak, Jonathan Chee, Alison M McDonnell

**Affiliations:** Institute for Respiratory Health, National Centre for Asbestos Related Diseases, University of Western Australia, Perth, WA, 6000, Australia; School of Biomedical Sciences, University of Western Australia, Crawley, WA, 6009, Australia; Institute for Respiratory Health, National Centre for Asbestos Related Diseases, University of Western Australia, Perth, WA, 6000, Australia; Institute for Respiratory Health, National Centre for Asbestos Related Diseases, University of Western Australia, Perth, WA, 6000, Australia; Colorectal Cancer Research Unit, St John of God Subiaco Hospital, Subiaco, WA, 6008, Australia; Medical School, University of Western Australia, Crawley, WA, 6009, Australia; Institute for Respiratory Health, National Centre for Asbestos Related Diseases, University of Western Australia, Perth, WA, 6000, Australia; Medical School, University of Western Australia, Crawley, WA, 6009, Australia; Institute for Respiratory Health, National Centre for Asbestos Related Diseases, University of Western Australia, Perth, WA, 6000, Australia; School of Biomedical Sciences, University of Western Australia, Crawley, WA, 6009, Australia; IIID Laboratory, Centre for Molecular Medicine and Innovative Therapeutics, Murdoch University, Murdoch, WA, 6150, Australia; IIID Laboratory, Centre for Molecular Medicine and Innovative Therapeutics, Murdoch University, Murdoch, WA, 6150, Australia; Institute for Respiratory Health, National Centre for Asbestos Related Diseases, University of Western Australia, Perth, WA, 6000, Australia; Medical School, University of Western Australia, Crawley, WA, 6009, Australia; Department of Respiratory Medicine, Sir Charles Gairdner Hospital, Perth, WA, 6000, Australia; Institute for Respiratory Health, National Centre for Asbestos Related Diseases, University of Western Australia, Perth, WA, 6000, Australia; School of Biomedical Sciences, University of Western Australia, Crawley, WA, 6009, Australia; Department of Respiratory Medicine, Sir Charles Gairdner Hospital, Perth, WA, 6000, Australia; Department of Respiratory Medicine, Sir Charles Gairdner Hospital, Perth, WA, 6000, Australia; School of Biomedical Sciences, University of Western Australia, Crawley, WA, 6009, Australia; School of Biomedical Sciences, University of Western Australia, Crawley, WA, 6009, Australia; The Kids Research Institute Australia, Perth, WA, 6000, Australia; Institute for Respiratory Health, National Centre for Asbestos Related Diseases, University of Western Australia, Perth, WA, 6000, Australia; School of Biomedical Sciences, University of Western Australia, Crawley, WA, 6009, Australia; Institute for Respiratory Health, National Centre for Asbestos Related Diseases, University of Western Australia, Perth, WA, 6000, Australia; Medical School, University of Western Australia, Crawley, WA, 6009, Australia; Department of Medical Oncology, Sir Charles Gairdner Hospital, Perth, WA, 6000, Australia; Institute for Respiratory Health, National Centre for Asbestos Related Diseases, University of Western Australia, Perth, WA, 6000, Australia; School of Biomedical Sciences, University of Western Australia, Crawley, WA, 6009, Australia; School of Biomedical Sciences, University of Western Australia, Crawley, WA, 6009, Australia; The Kids Research Institute Australia, Perth, WA, 6000, Australia

**Keywords:** malignant pleural effusion, mesothelioma, CD8^+^ T cells, TCR sequencing, tissue resident memory T cells

## Abstract

The success of cancer immunotherapies has highlighted the importance of monitoring the anti-tumour T cell response. Patients with mesothelioma frequently present with a malignant pleural effusion (MPE) that is commonly drained regularly to alleviate symptoms. As MPE contains tumour cells, T cells and cytokines, it provides a unique opportunity to sample immune events at the tumour site. However, there is minimal information on how MPE T cells are distinct from those in the blood, and whether T cell phenotypes unique to each compartment correlate with survival. We characterised T cell populations of matched MPE and blood from 31 mesothelioma patients using flow cytometry and bulk T cell receptor beta (TCRβ) sequencing. MPE CD8^+^ and CD4^+^ T cells displayed increased expression of PD-1, TIGIT, LAG-3 and TIM-3 compared to blood, with co-expression of inhibitory receptors greatest on MPE CD8^+^ T cells with a tissue resident memory T cell phenotype (CD69^+^CD103^+^). CD8^+^ TCRβ repertoires displayed clonal overlap between MPE and blood, suggesting that a majority of T cells traffic between these compartments. Finally, we show that high expression of PD-1 on circulating CD4^+^ T cells is an independent prognostic factor for poor survival in this patient group. This work suggests that MPE T cell phenotypes differ from those in circulation, with blood-based T cell subsets more sensitive predictors of outcome in this study.

## Introduction

Mesothelioma is an aggressive asbestos-induced cancer originating from mesothelial cells lining the pleura, peritoneum or pericardium [1]. At diagnosis, over 90% of patients present with a malignant pleural effusion (MPE), which is an abnormal accumulation of fluid in the pleural space [2]. MPE comprises of tumour cells, soluble factors, cytokines and immune cells, with T cells being one of the predominant immune cell subsets [3, 4]. As the MPE is adjacent to the tumour, it is a unique peri-tumoural environment that could reflect the tumour microenvironment.

The success of antibodies blocking T cell inhibitory receptors, known as immune checkpoint blockade (ICB) has highlighted the need to better understand and monitor T cells in patients, to allow for patient specific drug selection and to identify immune biomarkers predictive of response to therapy. This is particularly important as early surrogate clinical indicators of improved outcomes, such as radiological response, may not be seen until after several cycles of therapy. Dual or single agent anti-CTLA-4 and anti-PD-1/anti-PD-L1 ICB in a first or second line setting have significantly improved survival of mesothelioma patients [5–10]. However long-term control of disease is uncommon, causing an urgent need to define the tumour-immune landscape in ICB to improve therapy efficacy.

As T cells are key immune cells that mediate tumour cell killing after ICB, research has focused on profiling tumour infiltrating lymphocytes (TILs) to correlate with ICB efficacy. Serial tumour biopsies from mesothelioma patients are challenging to collect, whilst MPE is often recurrent and routinely drained to alleviate symptoms [11]. Monitoring immune responses in the periphery is commonly used due to ease of collection [12]. However, it does not accurately reflect immunity in the tumour microenvironment [13]. As MPE is adjacent to the tumour, MPE could provide a more robust source of biomarkers than peripheral blood to track anti-tumour T cell responses over time.

Key TIL phenotypes correlate with prognosis across multiple cancer types including mesothelioma, however there are limited reports for T cells in the MPE. TILs from mesothelioma patients that co-express high levels of inhibitory receptors (IhRs) such as PD-1, TIM-3, and TIGIT are associated with a worse prognosis [14–16]. MPE T cells in mesothelioma have been reported to express these inhibitory receptors individually [14, 17, 18] but the level of co-expression and hence prognostic potential is unknown. Tumour infiltrating tissue resident memory CD8^+^ T cells (T_RM_) correlate with improved overall survival in melanoma [19] and lung cancer [20], and are present in mesothelioma tumour samples [16], however they have not been characterised in the MPE from mesothelioma. Lastly, there is limited knowledge on antigen specific T cells in MPE while this could be important since the breadth of antigen specific TILs, as measured by the number of unique TCR clonotypes, correlates with increased overall survival following chemo-immunotherapy for mesothelioma [7].

Here, we performed in-depth profiling of MPE T cell phenotypes and TCRβ usage and compared that to matched peripheral blood (PB) T cells in mesothelioma patients. We posit that due to the proximity of the MPE to the tumour site, MPE will have increased proportions of memory T cells and increased co-expression of inhibitory receptors (IhR) compared to their counterparts in the blood. We also investigated how T cell features in blood and/or MPE from treatment naïve patients correlated with overall survival.

## Methods

### Patient selection

Paired samples of peripheral blood (PB) and malignant pleural effusion (MPE) were obtained from 31 patients with pleural mesothelioma (Table 1). Participants in the study had a histologically or cytologically confirmed diagnosis of pleural mesothelioma, with any stage or duration of disease and the majority of patients had no prior treatment (*n* = 28). Effusions from patients with prior talc pleurodesis or pleural infection were excluded. Patients were enrolled between August 2014 and February 2018. Mesothelioma subtypes epithelioid-containing and sarcomatoid were diagnosed by effusion cytology and histology of tissue biopsy respectively. This study was approved by institutional Human Research Ethics Committees and all patients provided written informed consent for collection and analysis of biological samples.

**Table 1.** Patient characteristics

	N = 31	%
***Gender***		
Male	28	90
Female	3	10
***Median Age (range)***	68 (30–89)	
Subtype*		
Epithelioid containing	27	87
Sarcomatoid	3	10
Unspecified subtype	1	3
***Prior Treatment***	3	10
***MPE Drainage***		
Indwelling pleural catheter (IPC)	23	74
Thoracocentesis	8	26
***MPE median collection vol (mL) (range)***	415 (25–2000)	
***Median days from diagnosis to sample collecting (range)***	56 (3–1738)	
***Treatment***		
Chemotherapy alone	9	29
Radiotherapy alone	2	7
Clinical Trial alone	2	7
Combination	11	36
Nil	7	23
***Median days between treatment and sample collection (range)***		
Before MPE sample (n = 3)	235 (209–1727)	
Post MPE sample (n = 21)	42 (1–372)	

* Epithelioid containing subtype diagnosed from pleural effusion cytology and sarcomatoid subtype by histology.

### Sample collection and processing

Whole blood was collected into a BD K_2_EDTA Vacutainer^®^ (BD Diagnostics) and peripheral blood mononuclear cells (PBMC) isolated by Ficoll-Paque™ density gradient centrifugation according to manufacturer’s instructions. Ficoll density centrifugation was used to isolate MPE mononuclear cells as previously described [18]. Mononuclear cells isolated from blood and MPE were stored in liquid nitrogen until analysis.

### Flow cytometry

Thawed cells were stained with two flow cytometry panels outlined in Supplementary Table S1. Prior to surface antibody staining, samples were incubated with Fixable Viability Dye eFluor™780 (ThermoFisher) suspended in PBS for 20 minutes in the dark at room temperature (RT). Antibodies for surface staining were suspended in Brilliant Stain Buffer (BD Biosciences) and incubated on cells in the dark for 40 minutes at RT. Hanks Balanced Salt Solution (without Ca2^+^, Mg2^+^, or phenol red)(ThermoFisher) supplemented with 0.5 mM EDTA and 2% neonatal calf serum (NCS) was used to wash cells between incubations. Samples were then fixed and permeabilized for 20 minutes in the dark at RT using the Foxp3/Transcription Factor Staining Buffer Set (eBioscience). Cells were washed with Permeabilization Buffer (eBioscience) and subjected to intracellular staining for 40 minutes in the dark at RT. Samples were washed and stored at 4°C in 1x stabilizing fixative (BD Biosciences). Samples were acquired on the BD LSRFortessa™ SORP (BD Biosciences) using BD FACS Diva software version 8.0.1 (BD Biosciences). All flow cytometry analyses were completed using FlowJo™ Software version 10 (BD Biosciences). Antibody concentrations and gating strategies are outlined (Supplementary Table S1, Supplementary Fig. S1A).

### Fluorescent activated cell sorting

Blood and MPE samples from 11 patients were stained for fluorescence activated cell sorting using the BD FACSAria™ cell sorter (BD Biosciences). All samples were stained with antibodies outlined in Supplementary Table S2. CD8^+^PD-1^+^, CD8^+^PD-1^−^, CD4^+^PD-1^+^ and CD4^+^PD-1^−^ T cells were sorted at > 80% efficiency for downstream bulk TCRβ sequencing. Sorted cells were collected in RNA protect cell reagent (QIAGEN) and stored at −20°C. Sorting gates are described in Supplementary Fig. S1J.

### Bulk TCRβ sequencing

TCRβ libraries were prepared as previously described in [21]. Briefly, total RNA was transcribed to cDNA adding unique molecular identifiers (UMI) for unbiased PCR amplification. The TCRβ locus was amplified by nested PCR using multiplexed V- and J-gene primers with the final PCR adding sequencing adaptors and barcodes. Paired-end (2x300bp) high-throughput sequencing was performed using the Illumina MiSeq platform (Illumina, RRID: SCR_016379). Data processing, aggregation of UMIs and alignment of CDR3 sequences to the IMGT/V-QUEST reference genome [22] were performed using repertoire analysis software based on MIGEC [23] (RRID: SCR_016337) and MiXCR [24] (RRID: SCR_018725) pipelines. Only sequences with UMIs were aligned.

### TCRβ sequencing analysis

TCRβ libraries were analysed using functions in R (R Project for Statistical Computing, RRID: SCR_001905, v3.6.0). TCRβ clones were defined as > 2 sequences with the same VDJ gene and CDR3 amino acid sequence. CDR3 sequences that were less than 8 or greater than 20 amino acids in length, included a stop codon or a frameshift were defined as non-functional and were excluded from analysis. TCRβ repertoires were down sampled to 1500 or 2000 total TCRβ clones for CD8^+^ and CD4^+^ T cell subsets respectively to ensure comparability. Diversity evenness 50 (DE_50_) was calculated as a percentage of clones that make up the top 50% of the repertoire, divided by the total number of clones in the repertoire. DE_50_ represents diversity as a frequency of the repertoire, with low DE_50_ corresponding to each clone evenly distributed in the repertoire [25]. Shannon’s entropy was calculated by; $H=-\sum_{i=1}^{n}p_{i}\ln p_{i}$ where p_i_ is the proportion of sequence i relative to the total N sequences [26].

### Statistical analysis

Paired PB and MPE immune cell phenotype data are presented as before-after plots. Where the difference between each pair (PB and MPE) was normally distributed, comparisons were made using a paired *t*-test and results described as mean ± SD in the text. The Wilcoxon matched-pairs signed rank test (nonparametric paired *t*-test) was used for comparisons where the difference between pairs was not normally distributed and results described as median[interquartile range (IQR)]. For unpaired comparisons, normally distributed variables are presented as mean ± SD and compared using t-tests. The nonparametric Kruskal-Wallis test followed by the Dunn’s multiple comparisons was used where variables were not normally distributed. All statistics were performed using GraphPad Prism Software (Graph Pad Software Inc., v10). Results were considered significant when *P* < 0.05 (*P < 0.05, **P < 0.01, ***P < 0.001, ****P < 0.0001). Exploratory analysis for correlation of immune cell phenotypes with overall survival (OS) was performed in R (version 4.3.0) using the survival (3.5-5), survivalAnalysis (0.3.0) and olsrr (0.5.3) packages. Overall survival, defined as time from sample collection until death from any cause, was the endpoint used for survival analyses. Surviving patients were censored at date last seen alive (last clinical follow-up) prior to censoring (31^st^ December 2018). Immunological variables were first assessed as continuous variables and hazard ratios calculated using univariate Cox regression. Categorical variables were then created for each immune biomarker with a *P* < 0.05 using data driven dichotomization [27]. Kaplan-Meier survival curves were compared using the log-rank test and hazard ratios (HR) were calculated using univariate Cox regression. All variables with a *P* < 0.05 were included in the multivariate model, with survival time used as a continuous outcome. Backward stepwise Cox regression was used to determine the final model with variables *P* < 0.05 remaining in the model at each step.

## Results

### Patient and sample characteristics

We analysed peripheral blood (PB) and malignant pleural effusion (MPE) T cells from 31 patients with pleural mesothelioma (Table 1). Epithelioid was the predominant histological subtype (*n* = 27; 87%). Three patients (10%) had sarcomatoid, one (3%) was not identified. MPE was drained by an indwelling pleural catheter for most patients (*n* = 23, 74%), while the remaining were drained by thoracocentesis (*n* = 8, 25%). With the exception of three patients, samples were collected prior to any treatment. Following sample collection, patients received a range of treatments with 29% receiving chemotherapy alone, 7% receiving radiotherapy, 7% selected for a clinical trial, 36% received combination therapy and the remaining receiving no treatment (Table 1).

### Malignant pleural effusions have increased Tregs and central memory T cells compared to matched peripheral blood

**Figure 1.**
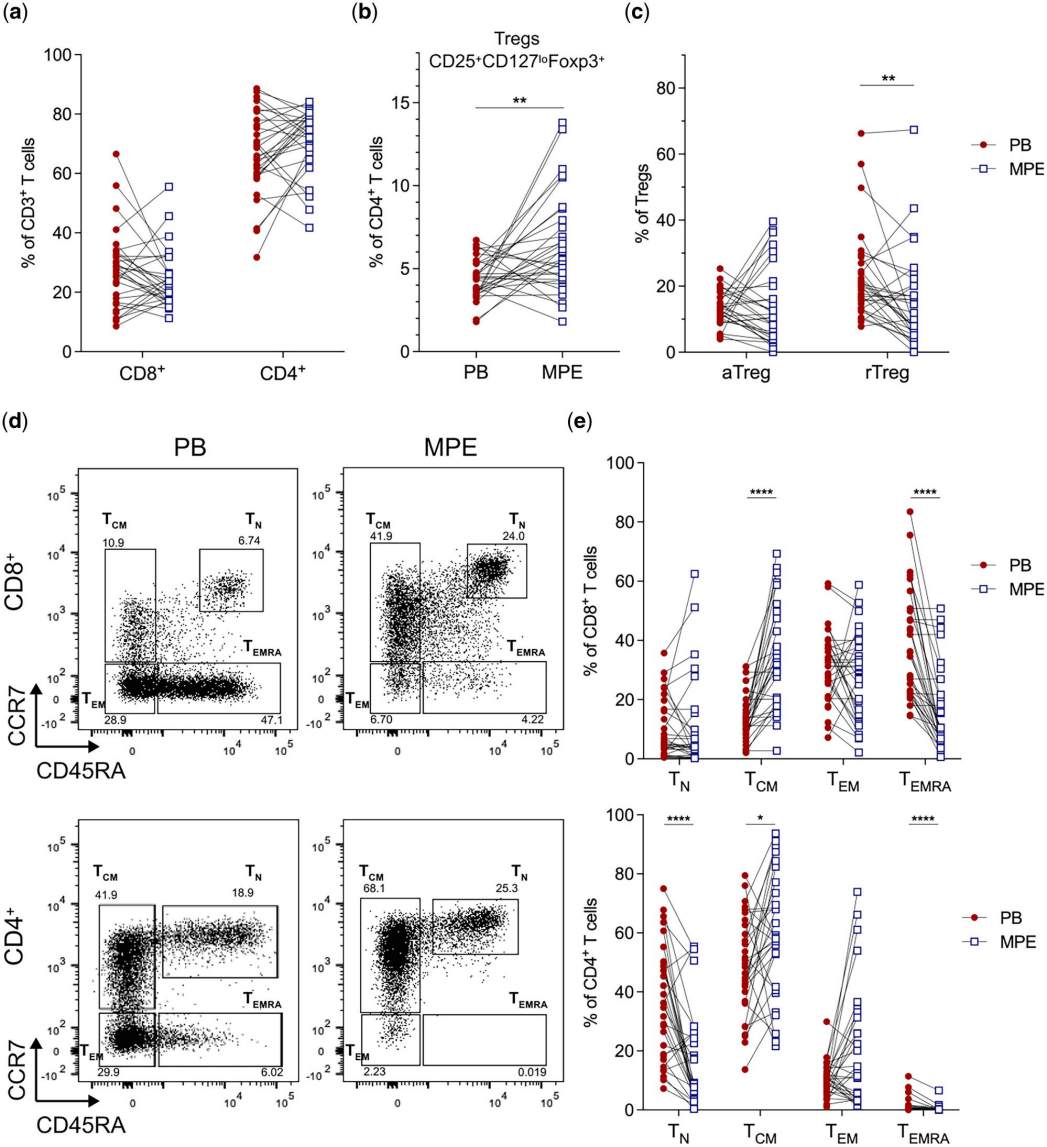
Regulatory T cells (Tregs) and central memory T cells were increased in MPE compared to matched PB in mesothelioma patients. (a) Frequency of total CD4^+^ and CD8^+^ T cells in matched blood (PB) and pleural effusion (MPE). (b) Frequency of total Tregs, (c) activated (aTregs; Foxp3^hi^CD45RA^-^) and resting (rTregs; Foxp3^lo^CD45RA^+^) in matched PB and MPE. (d) Representative plots displaying memory subsets of CD8^+^ (top) and CD4^+^ (bottom) T cells in the matched PB (left) and MPE (right). (e) Proportion of naïve (T_N_; CCR7^+^CD45RA^+^), central memory (T_CM_; CCR7^+^CD45RA^−^), effector memory (T_EM_; CCR7^-^CD45RA^-^) and terminally differentiated (T_EMRA_; CCR7^−^CD45RA^+^) CD8^+^and CD4^+^ T cells in PB and MPE. Lines connecting PB and MPE samples represent samples obtained from the same patient. Paired *t*-test was used for normally distributed data, otherwise Wilcoxon matched-pairs signed-rank test was used. *P < 0.05, **P < 0.01, ****P < 0.0001.

As tumours are infiltrated by different T cells subsets including regulatory CD4^+^ T cells (Tregs) and memory CD8^+^ T cells [28], we first compared the phenotype of T cell populations in paired blood and MPE samples. The overall proportions of CD8^+^ and CD4^+^ T cells were similar between the blood and MPE (Fig. 1a). MPE had significantly greater proportions of regulatory T cells (Tregs; CD4^+^CD25^+^CD127^lo^Foxp3^+^) compared to the blood (Fig. 1b). Specifically, the proportion of resting Tregs (rTregs; Foxp3^lo^CD45RA^+^) were significantly decreased in the MPE compared to the blood [29], whereas activated Tregs (aTregs; FoxP3^hi^CD45RA^-^) were similar between compartments (Fig. 1c).

We next characterised T cell memory subsets [30]. MPE had significantly greater proportions of central memory (T_CM_) CD8^+^ and CD4^+^ T cells compared to matched blood (Fig. 1d and e). In contrast, the frequency of terminally differentiated effector memory (T_EMRA_) CD8^+^ and CD4^+^ T cells were significantly decreased in the MPE. The blood had a significantly greater frequency of naïve CD4^+^ T cells (T_N_) compared to matched MPE. Proportions of CD8^+^ TN cells and effector memory (T_EM_) CD8^+^ and CD4^+^ T cells were similar between both compartments. Thus, MPE associated with pleural mesothelioma is enriched in Tregs, and central memory T cells, whereas CD8^+^ T_EMRA_ cells dominate the circulating T cell pool.

### T Cells have increased expression of inhibitory receptors in the malignant pleural effusion compared to the peripheral blood

**Figure 2.**
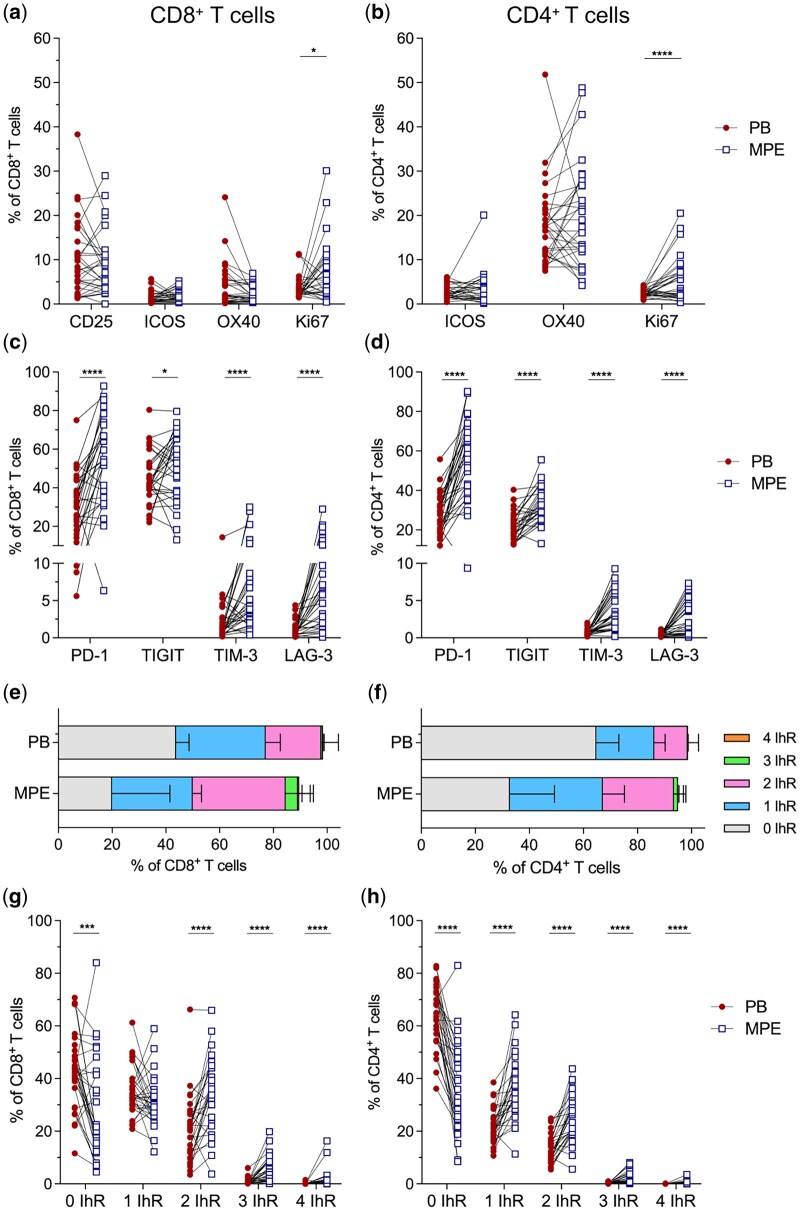
MPE T cells have increased co-expression of inhibitory receptors. (a and b) Frequency of activation markers; CD25, OX40, ICOS and Ki67 expressed on CD8^+^ and CD4^+^ T cells in the PB and MPE. (c and d) Frequency of inhibitory receptors (IhR): PD-1, TIGIT, TIM-3 and LAG-3 on CD8^+^ and CD4^+^ T cells in the PB and MPE. (e and f) Stacked bar plots displaying the proportion of CD8^+^ and CD4^+^ T cells expressing only 1 IhR, any 2 IhRs, any 3 IhRs or all 4 IhRs in PB and MPE. (g-h) Frequency of CD8^+^ and CD4^+^ T cells expressing 0–4 IhRs in matched PB and MPE samples. Lines connecting PB and MPE samples represent samples obtained from the same patient. Data presented as median [IQR] in E, F. Paired *t*-test was used for normally distributed data, otherwise Wilcoxon matched-pairs signed-rank test was used. *P < 0.05, **P < 0.01, ***P < 0.001, ****P < 0.0001.

To gain further insight into the activation status of T cells in both compartments, we compared the expression of T cell activation and inhibitory markers. MPE displayed a significantly greater proportion of CD8^+^ and CD4^+^ T cells expressing the proliferation marker Ki67 compared to the blood (Fig. 2a and b), suggesting proliferation of T cells within the MPE. The expression of activation markers CD25, ICOS and OX40 were similar between both compartments (Fig. 2a and b). MPE displayed a significantly increased frequency of Tregs expressing ICOS and OX40 compared to the blood (Supplementary Fig. S2A). The frequency of Ki67^+^ Tregs was similar between both compartments (Supplementary Fig. S2A).

Upregulation of inhibitory receptors (IhR) negatively regulates T cell activation. The expression of multiple IhRs often indicates chronic antigen exposure and is a feature of suppressed T cells within the tumour microenvironment. We analysed the expression of IhRs PD-1, TIGIT, TIM-3 and LAG-3 on CD8^+^ and CD4^+^ T cells. In the MPE, PD-1 was the predominant IhR expressed on both T cell subsets (Fig. 2c and d). The frequency of CD8^+^ and CD4^+^ T cells expressing PD-1 was significantly greater in the MPE compared to the blood. In addition, the proportion of CD8^+^ and CD4^+^ T cells expressing TIGIT, TIM-3 or LAG-3 was significantly greater in the MPE than the blood (Fig. 2c and d). Tregs also displayed a significantly increased expression of PD-1 and TIGIT in the MPE compared to the blood (Supplementary Fig. S2B). To characterise co-expression of IhRs, we grouped T cells expressing only 1, any 2, any 3 or all 4 IhRs (Supplementary Fig. S2C and D). The proportion of CD8^+^ and CD4^+^ T cells expressing either any 2 IhRs or any 3 IhRs was significantly increased in the MPE compared to the blood (Fig. 2e–h). CD8^+^ and CD4^+^ T cells expressing all 4 IhRs were increased in MPE compared to blood (Fig. 2g and h). Importantly, IhR co-expression on T cells always included PD-1 (Supplementary Fig. S2C–F). LAG-3 or TIM-3 were only expressed on T cells with PD-1, and the most abundant IhR combination for CD8^+^ and CD4^+^ T cells was PD-1 and TIGIT (Supplementary Fig. S2E and F). While T cells were proliferative in the MPE, they had co-expression of multiple inhibitory receptors, suggesting chronic antigen exposure and dysfunction, similar to T cells in the tumour microenvironment. In line with this, CD8^+^ T cells in the MPE were enriched for a GzB^+^PD-1^+^ phenotype, predominantly within the T_EM_ and T_CM_ subsets, suggesting sustained antigenic stimulation and potential functional dysregulation. Conversely, circulating CD8^+^ T cells were enriched for GzB^+^PD-1^−^ T_EMRA_ cells, indicative of terminally differentiated cytotoxic effectors with preserved function (Supplementary Fig. S2G and H).

### Expanded TCRβ clones from malignant pleural effusions are shared with the peripheral blood

**Figure 3.**
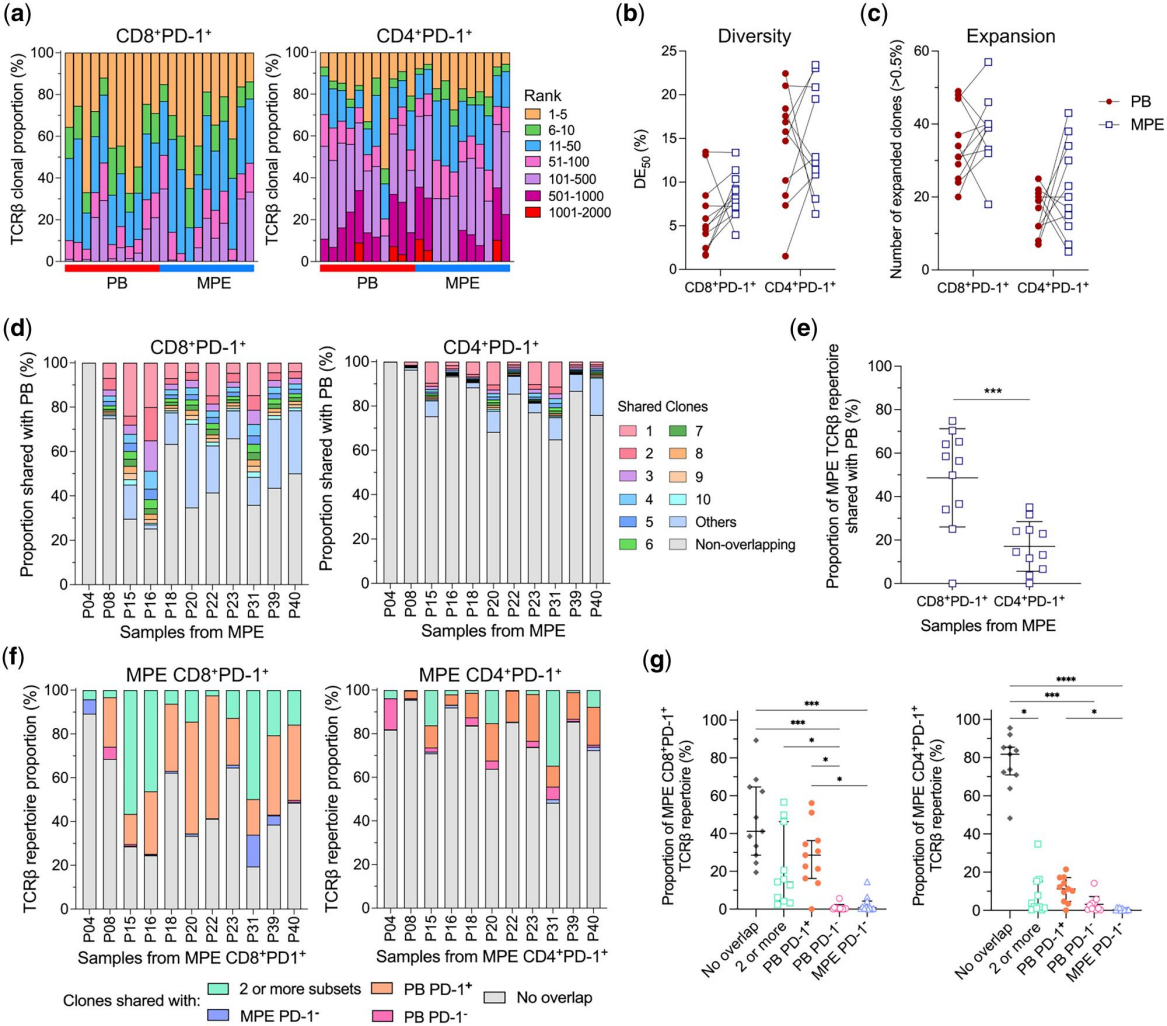
CD8^+^ and CD4^+^ TCRβ repertoires were similar between the MPE and PB, with shared clones in PD-1^+^ populations between compartments. (a) Distribution of TCRβ clones, ranked by total abundance in CD8^+^PD-1^+^ (left) and CD4^+^PD-1^+^ (right) TCRβ repertoires. Each bar represents the repertoire for each patient sample. (b) Diversity evenness (DE_50_) of TCRβ repertoires between paired PB and MPE for each TCRβ repertoire. Lower DE_50_ indicates a less even, more clonal repertoire. Lines connecting PB and MPE samples represent samples obtained from the same patient. (c) Proportion of expanded clones that make up greater than 0.5% of each TCRβ repertoire. (d) Bar plots of MPE CD8^+^PD-1^+^ (left) and CD4^+^PD-1^+^ (right) TCRβ repertoires, coloured by the proportion of most abundant, shared TCRβ clones that are also present in matched PB samples. Each bar represents sharing from matched samples of each patient. (e) Proportion of clones in MPE CD8^+^PD-1^+^ and CD4^+^PD-1^+^ TCRβ repertoires also found in paired PB. Data presented as mean ± SD. (f) Bar plots of MPE CD8^+^PD-1^+^ (left) and CD4^+^PD-1^+^ (right) TCRβ repertoires, coloured by the proportion of TCRβ clones that are found exclusively or collectively in PB PD-1^+^, PB PD-1^−^ and MPE PD-1^−^ TCRβ repertoires for each patient. (g) Dot plots representing proportion of MPE CD8^+^PD-1^+^ (left) and CD4^+^PD-1^+^ (right) TCRβ repertoire that are non-overlapping, shared between 2 or more T cell subsets, only with PB PD-1^+^, PB PD-1^−^ or MPE PD-1^−^ TCRβ repertoires. Data presented as median [IQR]. Paired *t*-test was used for normally distributed data, otherwise Wilcoxon matched-pairs signed-rank test was used. Kruskal-Wallis test with Dunn’s multiple comparisons was used to compare between 4 groups. *P < 0.05, **P < 0.01, ***P < 0.001, ****P < 0.0001.

T cell activation is often accompanied by clonal expansion and activation of antigen-specific T cells. As MPE contained increased frequencies of activated T cells, we hypothesized that oligoclonal T cell expansion would be restricted to a few dominant clones in the MPE. In contrast, clonal expansion would occur to a lesser extent in the blood. As PD-1 was the most abundantly expressed IhR in the MPE, and is a known marker for antigen experienced T cells [31], we characterised the TCRβ repertoire of CD8^+^PD-1^+^, CD4^+^PD-1^+^, CD8^+^PD-1^−^ and CD4^+^PD-1^−^ T cells collected from 11 patients in the cohort, comparing-between matched blood and MPE. The number of sorted T cells, total TCRβ sequences and unique TCRβ sequences were similar between the blood and MPE, except for CD4^+^PD-1^−^ MPE T cells which had fewer unique TCRβ clones compared to blood (Supplementary Fig. S3A). Oligoclonal expansion was observed in both the blood and MPE regardless of PD-1 status, as the top 5 most abundant clones from CD8^+^PD-1^+^ populations made up 30.8 ± 4.81% of total clones in the MPE, and 39.4 ± 5.42% in the blood (Fig. 3a). For CD8^+^PD-1^−^, CD4^+^PD-1^+^ and CD4^+^PD-1^−^ cells, the top 5 most clones accounted for 48.5 ± 5.54%/41.4 ± 8.00%, 19.6 ± 4.19%/14.7 ± 2.24% and 13.0 ± 2.67%/22.7 ± 4.02% in MPE/blood respectively (Fig. 3a, Supplementary Fig. S3B). Of all the cell subsets, only CD4^+^PD-1^−^ T cells from MPE had a significantly higher proportion of the most abundant clones (ranks 1–5, and 11–50) compared to their blood counterparts (Supplementary Fig. S3C). Each sample was further quantified with different clonality and diversity metrics, as each metric captures different features of the TCRβ repertoire structure. The diversity evenness 50 (DE_50_) score measures the number of unique TCRβ clones that account for 50% of a sample’s TCRβ repertoire. A lower DE_50_ score reflects reduced evenness, and skewing towards abundant clones within a sample [25]. DE_50_ scores were similar between the blood and MPE for both CD8^+^PD-1^+^ and CD4^+^PD-1^+^ T cells (Fig. 3b). DE_50_ of CD8^+^PD-1^−^ T cells was lower in blood compared to MPE (Supplementary Fig. S3D). In line with the proportions of ranked clones (Supplementary Fig. S3C), DE_50_ of CD4^+^PD-1^−^ T cells was lower in MPE compared to blood (Supplementary Fig. S3D). Shannon’s entropy measures the overall diversity of the whole repertoire, taking into account both rare and dominant clones. A lower Shannon’s entropy is reflective of reduced diversity and a more imbalanced clonal distribution. Shannon’s entropy was similar between MPE and blood T cells regardless of PD-1 expression and CD4/8 subsets (Supplementary Fig. S3E). This was also the case with the number of highly expanded clones, defined as those that occupy more than 0.5% of the TCRβ repertoire (Fig. 3c and Supplementary Fig. S3E). CD4^+^PD-1^−^ TCRβ repertoire was the only population to show consistent differences between blood and MPE across two metrics. These differences are likely driven by expansion of intermediate-frequency clones in the MPE.

We next examined the clonal overlap between the MPE and blood. If clonal expansion in the MPE was occurring locally due to antigens unique to MPE, we posit that there would be minimal TCRβ overlap of PD-1^+^ T cells between both MPE and blood. 48.6 ± 22.6% of CD8 + PD-1^+^ TCRβ clones in the MPE samples were present in paired CD8^+^PD-1^+^ blood samples (Fig. 3d and e). Likewise, 16.6 ± 11.1% MPE CD4^+^PD-1^+^ TCRβ clones were also found in the CD4^+^PD-1^+^ blood samples, but to a lesser degree than their CD8^+^ counterparts (Fig. 3e). This was also the case with PD-1^-^ T cells, as 32.1 ± 23.8% of CD8^+^PD-1^-^ TCRβ clones, and 15.2 ± 20.0% of CD4^+^PD-1^−^ TCRβ clones in the MPE were present in paired blood samples (Supplementary Fig. S3F, G). We compared the frequencies of shared, unique clones in both MPE and blood, identifying clonotypes that were > 5-fold more abundant in one compartment. In the combined CD8^+^PD-1^+^ TCRβ repertoire, 91 of all 591 shared clones were enriched in MPE versus 63 in the blood (Supplementary Fig. S3H). Similarly, in the combined CD4^+^PD-1^+^ repertoire, 93 of all 750 shared clones were MPE-enriched compared to 60 in the blood (Supplementary Fig. S3H). Combined CD8^+^PD-1^-^ TCRβ repertoires showed a balanced distribution (34 versus. 32 out of 291 clones, MPE versus. PB), whereas CD4^+^PD-1^−^ TCRβ repertoires had more MPE-enriched clones (49 versus. 16 out of 252 clones, MPE versus. PB) (Supplementary Fig. S3H). Many CD8^+^ T cell clones are shared between MPE and blood, with preferential expansion of shared CD8^+^PD-1^+^ T cell clones in the MPE. CD4^+^ T cell clones displayed less overlap and were more compartmentalised within the MPE.

To investigate if local differentiation of T cells (conversion between PD-1^-^ and PD-1^+^ T cells) could be driving the preferential clonal expansion in the MPE, we compared overlap of clones between CD8^+^PD-1^-^ and CD8^+^PD-1^+^ T cells from the MPE, and performed similar comparison between these subsets in blood (Fig. 3f). The proportion of MPE CD8^+^PD-1^+^ TCRβ repertoire that overlapped with MPE CD8^+^PD-1^-^ TCRβ repertoire was significantly lower than those overlapped with CD8^+^PD-1^+^, CD8^+^PD-1^-^ TCRβ repertoires in the blood (Fig. 3g), suggesting that local differentiation between PD-1^-^ and PD-1^+^CD8^+^ T cells in the MPE was minimal, and unlikely to be contributing to clonal expansion. Most of the MPE CD4^+^PD-1^+^ TCRβ repertoire did not overlap with other PD-1 subsets in blood or MPE (Fig. 3f and g). Taken together, these data indicate overlap between blood and MPE, particularly for CD8^+^ T cells, suggesting trafficking of T cells between the two compartments. MPE CD4^+^ T cell clones were highly unique to each compartment, suggesting local retention and minimal trafficking.

### Tissue resident memory T cells (T_RMs_) are detected in the MPE, with greater co-expression of inhibitory receptors compared to other memory T cell subsets

**Figure 4.**
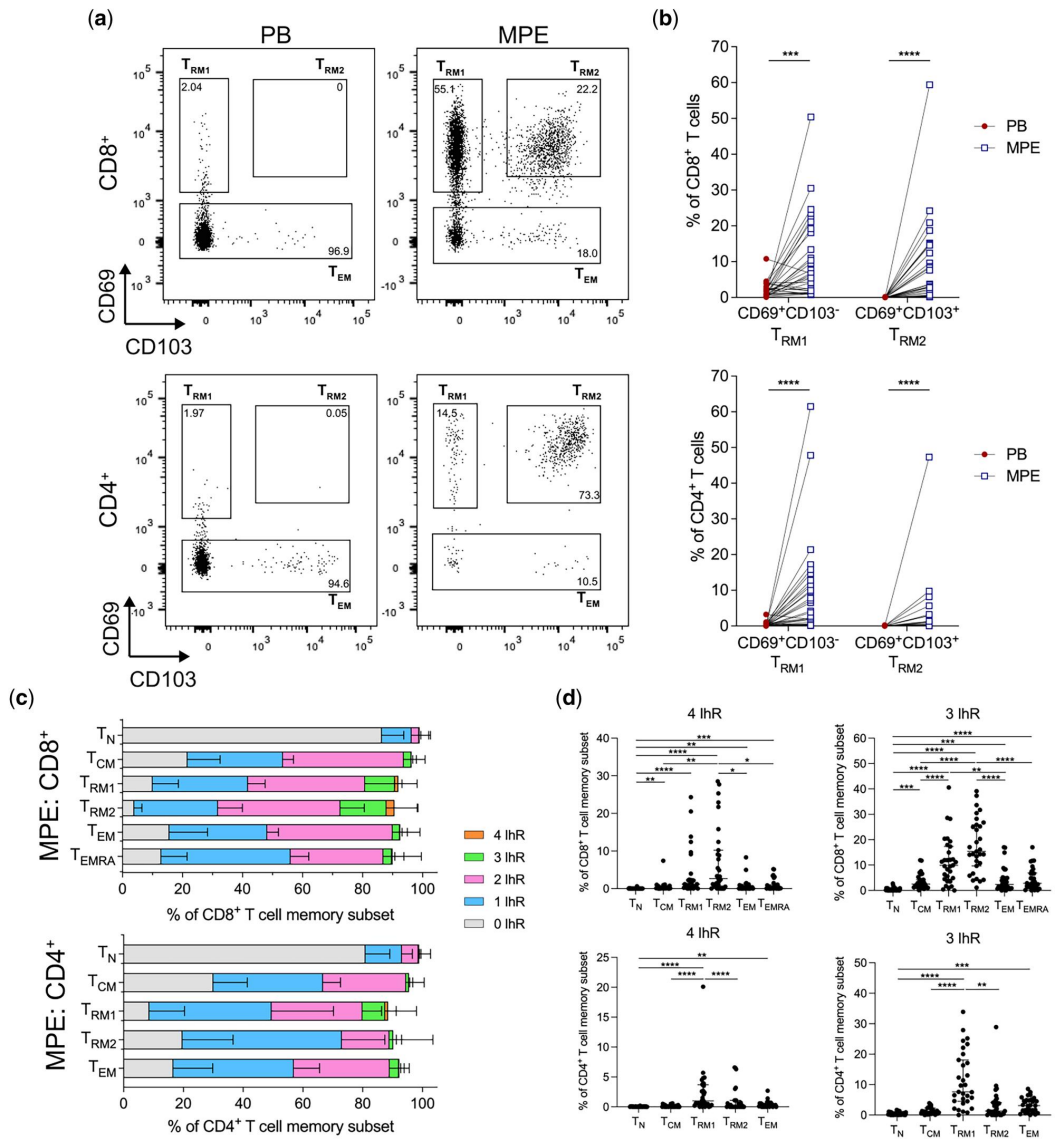
MPEs were enriched with tissue resident memory T cells (T_RM_) that exclusively co-express inhibitory receptors. (a) Representative FACs plots representing T_RM1_, T_RM2_ and T_EM_ subsets by CD69 and CD103 expression of CCR7^+^CD45RA^−^ CD8^+^ (top) or CD4^+^ (bottom) T cells in the PB (left) and MPE (right). (b) Frequency of T_RM1_ and T_RM2_ CD8^+^ and CD4^+^ T cells in the PB and MPE. (c) Stacked bar plots displaying proportion of CD8^+^ (top) and CD4^+^ (bottom) memory T cell subsets that co-expressed 1 IhR, any 2 IhRs, any 3 IhRs or all 4 IhRs in the MPE. (d) Frequency of CD8^+^ (top) and CD4^+^ (bottom) subsets expressing all 4 IhRs (left) or any 3 IhRs (right). The number of MPE CD4^+^ T_EMRA_ was too low for accurate phenotypic analysis. Data presented as median [IQR] in (c, d). Paired *t*-test was used for normally distributed data, otherwise Wilcoxon matched-pairs signed-rank test was used. Kruskal-Wallis test with Dunn’s multiple comparisons was used to compare between 5 or 6 groups. *P < 0.05, **P < 0.01, ***P < 0.001, ****P < 0.0001.

Tissue resident memory T cells (T_RMs_) have emerged as key players in the anti-tumour immune response, particularly for their critical role in mediating efficacy of ICB [32]. T_RMs_ are characterised by CD69, CD103 and lack of expression of the lymph node chemokine receptor CCR7, causing their residence in tissues. We queried whether we could find a T cell subset unique to MPE based on tissue residency. Using CD69 and CD103 expression as a guide, we characterised two T_RM_-like populations from the T_EM_ (CCR7^-^CD45RA^−^) population of CD8^+^ and CD4^+^ T cells in paired blood and MPE (Fig. 4a). We found that both T_RM_ subsets; T_RM1_ (CD69^+^CD103^−^) and T_RM2_ (CD69^+^CD103^+^) were restricted to the MPE. CD8^+^ and CD4^+^ T_RM1_ and T_RM2_ cells were significantly greater in the MPE than the paired blood (Fig. 4b).

As IhRs were highly expressed on T cells in the MPE compared to the blood (Fig. 2), we characterised the expression of IhRs on MPE memory subsets, including T_RMs_. We found that the co-expression of all four inhibitory receptors; PD-1, TIGIT, TIM3 and LAG-3, that was restricted to the MPE, were present on T_RM_ subsets (Fig. 4c and d, Supplementary Fig. S4A–C). CD8^+^ T_RM1_ cells expressed a significantly greater frequency of all 4 or any 3 IhRs compared to CD8^+^ TN, and T_CM_ cells. CD8^+^ T_RM2_ cells expressed a significantly greater frequency of all 4 or any 3 IhRs compared to CD8^+^ T_N_, T_CM_, T_EM_, T_EMRA_ cells (Fig. 4c and d, Supplementary Fig. S4A). CD4^+^ T_RM1_ cells displayed co-expression of all 4 or any 3 IhRs compared to CD4^+^ T_RM2_, CD4^+^ T_N_ and T_CM_ cells (Fig. 4c and d). The most abundant IhR combination on MPE T_RM1_ and T_RM2_ was the co-expression of PD-1 and TIGIT (Supplementary Fig. S4C). There was no difference in co-expression of IhRs on CD8^+^ and CD4^+^ T_N_, T_CM_, T_EM_, T_EMRA_ cells in the periphery (Supplementary Fig. S4D). Together, these data show that T_RMs_ are present in the peri-tumoural environment and are characterised by co-expression of multiple inhibitory receptors.

### Increased proportions of CD4 + PD-1 + T cells in the periphery associate with poor survival

Finally, we sought to determine the prognostic significance of the observed T cell phenotypes in the MPE and blood. Baseline proliferating CD8^+^ T cells in the periphery have previously been shown to predict for survival in patients with mesothelioma and non-small cell lung cancer [33]. Given the prognostic significance of TILs in many types of cancer, including mesothelioma, we hypothesized that T cell phenotypes within tumour associated MPE would be more sensitive predictors of prognosis than those in circulation. Baseline clinical variables previously shown to have prognostic value in patients with mesothelioma, including age, histology, white blood cell count (WBC), platelet count, hemoglobin, neutrophil to lymphocyte ratio and BRIMS scores [34], were assessed in univariate Cox regression models. Clinical variables found to be associated with poor prognosis in this patient population were increased age and platelet count at diagnosis (Table 2). The number of T cell phenotypes significantly associated with overall survival was higher in the blood than MPE (Table 2, Supplementary Table S3). Circulating naïve CD8^+^ T cells, CD8^+^ T_EMRA_, CD4^+^ PD-1^+^ T cells, CD4^+^ TIGIT^+^ T cells and CD4^+^ T cells co-expressing both PD-1 and TIGIT (CD4^+^ PD-1^+^ TIGIT^+^ T cells) were significantly associated with overall survival. In contrast, only three T cell phenotypes were significant in the MPE; CD8^+^ T_EMRA,_ proliferating CD4^+^ T cells (Ki67^+^) and CD4^+^ PD-1^+^ TIGIT^+^ T cells were prognostic in this patient group. All T cell phenotypes significantly associated with overall survival as continuous variables were also significant following data driven dichotomization. Patients with increased proportions of circulating naive CD8^+^ T cells (>6.78% of total CD8^+^ T cells) had a median survival of 19 months, whereas the reciprocal group (<6.78%) had a substantially shorter median survival of 6 months (Supplementary Fig. S5). In contrast, increased frequencies of circulating CD8^+^ T_EMRA_ cells, CD4^+^ PD1^+^ T cells, CD4^+^TIGIT^+^ T cells and CD4^+^ PD-1^+^TIGIT^+^ T cells and MPE-derived CD8^+^ T_EMRA_ cells, CD4^+^ Ki67^+^ T cells and CD4^+^ PD1^+^TIGIT^+^ T cells were all negatively associated with overall survival (Supplementary Fig. S5). There was no significant correlation between TCRβ repertoire diversity in blood or MPE, as measured by DE_50_, with overall survival (Supplementary Fig. S6).

**Table 2.** Univariate cox regression for clinical and immunological variables

	HR	95% CI	*p*-value
***Clinical Variables***			
**Age**	**1.09**	**1.03–1.15**	**0.002**
Sex (Male/Female)	1.53	0.34–6.80	0.577
WBC (x10^9^/L)	1.24	0.94–1.64	0.123
Subtype (Epithelioid-containing, sarcomatoid)	2.38	0.29–19.42	0.418
**Platelet count (x10^9^/L)**	**1.01**	**1.00–1.01**	**0.020**
NLR	1.12	0.93–1.34	0.229
Albumin (g/L)	0.93	0.83–1.04	0.191
Hemoglobin (g/L)	0.98	0.95–1.00	0.064
BRIMS Score [1–4, 34]	1.74	0.73–4.16	0.215
***Peripheral Blood Variables***			
% CD4^+^ Treg cells	1.01	0.95–1.07	0.821
% CD8^+^ Ki67^+^ T cells	1.06	0.91–1.23	0.451
% CD4^+^ Ki67^+^ T cells	1.56	0.91–2.67	0.105
**% CD8^+^ T_N_ cells**	**0.91**	**0.85–0.98**	**0.012**
**% CD8^+^ T_EMRA_ cells**	**1.03**	**1.01–1.06**	**0.006**
**% CD4^+^ PD-1^+^ T cells**	**1.05**	**1.00–1.10**	**0.041**
**% CD4^+^ TIGIT^+^ T cells**	**1.10**	**1.02–1.19**	**0.017**
**% CD4^+^ PD-1^+^ TIGIT^+^ T cells**	**1.14**	**1.03–1.26**	**0.013**
% CD8^+^ PD-1^+^ T cells	0.97	0.94–1.01	0.114
***MPE Variables***			
% CD4^+^ Treg cells	1.01	0.79–1.30	0.933
% CD8^+^ Ki67^+^ T cells	1.05	0.98–1.13	0.147
**% CD4^+^ Ki67^+^ T cells**	**1.11**	**1.01–1.21**	**0.025**
% CD8 T_N_ cells	0.97	0.94–1.01	0.172
% CD8^+^ T_RM1_ cells	1.00	0.95–1.05	0.986
% CD8^+^ T_RM2_ cells	1.01	0.97–1.05	0.555
**% CD8^+^ T_EMRA_ cells**	**1.04**	**1.01–1.07**	**0.018**
% CD4^+^ PD-1^+^ T cells	1.02	1.00–1.04	0.116
% CD4^+^ TIGIT^+^ T cells	1.05	1.00–1.10	0.079
**% CD4^+^ PD-1^+^ TIGIT^+^ T cells**	**1.06**	**1.00–1.12**	**0.036**
% CD8^+^ PD-1^+^ T cells	1.00	0.98–1.02	0.937

All immunological variables were assessed as continuous variables. Categorical variables (Sex and BRIMS Score) were transformed into numeric codes before entering the model. Patients with BRIMS score 1 and 2 were combined (*n* = 15) and compared with BRIMS score 3 and 4 (*n* = 16). Bold values denote significant associations (*P* < 0.05). CD4^+^ Tregs defined as CD4^+^CD127^lo^CD25^hi^Foxp3^+^ cells. *HR* hazard ratio, *CI* confidence interval, *WBC* white blood cell count, *MPE* malignant pleural effusion, *NLR* neutrophil to lymphocyte ratio, *T_N_* naïve T cells (CD45RA^+^CCR7^+^), *T_CM_* central memory T cells (CD45RA^-^CCR7^+^), *T_EM_* effector memory T cells (CD45RA^-^CCR7^−^), *T_EMRA_* terminally differentiated memory T cells (CD45RA^+^CCR7^−^), *T_RM1_* tissue resident memory T cells subset 1 (CD69^+^CD103^−^), *T_RM2_* tissue resident memory T cells subset 2 (CD69^+^CD103^+^).

Multivariate backward stepwise Cox regression analysis was then used to assess independent association of clinical variables and T cell phenotypes significantly associated with overall survival on univariate analysis. Two multivariate models were tested, both of which included age at diagnosis, platelet count at diagnosis, proportion of blood CD8^+^ T_N_ cells, MPE CD4^+^ Ki67^+^ T cells, MPE CD4^+^ PD-1^+^TIGIT^+^ T cells and blood and MPE CD8^+^ T_EMRA_ cells. To avoid mathematical coupling [35], one of the multivariate models included individual expression of PD-1 and TIGIT by CD4^+^ T cells in blood (Model 1; CD4^+^ PD1^+^ T cells, CD4^+^ TIGIT^+^ T cells) and the other, co-expression of PD-1 and TIGIT by blood CD4^+^ T cells (Model 2: CD4^+^ PD-1^+^ TIGIT^+^ T cells) (Table 3, Supplementary Table S4). In model 1, age at diagnosis, platelet count and proportion of PD-1 expressing CD4^+^ T cells in peripheral blood (PB % CD4^+^ PD-1^+^ T cells) were independent prognostic factors for poor survival. In model 2, age at diagnosis, platelet count and the proportion of circulating CD4^+^ T cells co-expressing both PD-1 and TIGIT (PB % CD4^+^ PD-1^+^TIGIT^+^ T cells) were independent prognostic factors for poor overall survival. Together, these data suggest that while multiple T cells subsets in the blood and MPE were associated with survival, only the proportion of circulating CD4^+^ T cells expressing PD-1 was independently prognostic in this group.

**Table 3.** Multivariate cox regression for variables significantly associated with survival in univariate analysis.

Variables	β coefficient (SE)	t-value	*p*-value
***Model 1***			
Age	−0.480 (0.170)	−2.815	0.009
Platelet count (x10^9^/L)	−0.052 (0.018)	−2.950	0.007
PB % CD4^+^ PD-1^+^ T cells	−0.568 (0.188)	−3.021	0.006
***Model 2***			
Age at sample collection	−0.416 (0.174)	−2.394	0.024
Platelet count (x10^9^/L)	−0.051 (0.017)	−2.913	0.007
PB % CD4^+^ PD-1^+^ TIGIT^+^ T cells	−1.132 (0.363)	−3.114	0.004

Model 1 (age at diagnosis, platelet count, % PB CD8^+^ T_N_ cells, % PB CD8^+^ T_EMRA_ cells, % PB CD4^+^PD-1^+^, % PB CD4^+^TIGIT^+^ T cells, % MPE CD4^+^Ki67^+^ T cells, % MPE CD4^+^PD-1^+^TIGIT^+^, % MPE CD8^+^ T_EMRA_ cells): F statistic = 10.07, p-value = 0.00015, R^2^ = 0.537, Adjusted R^2^ = 0.484.

Model 2 (age at diagnosis, platelet count, % PB CD8^+^ T_N_ cells, % PB CD8^+^ T_EMRA_ cells, % PB CD4^+^PD-1^+^TIGIT^+^, % MPE CD4^+^Ki67^+^ T cells, % MPE CD4^+^PD-1^+^TIGIT^+^, % MPE CD8^+^ T_EMRA_ cells): F statistic = 10.37, p-value = 0.00012, R^2^ = 0.545, Adjusted R^2^ = 0.492.

*PB* peripheral blood, *SE* standard error.

## Discussion

Here, we performed in-depth T cell profiling of matched MPE and blood from mesothelioma patients and found that surface expression of receptors associated with T cell memory, exhaustion, and tissue-residence distinguished MPE from blood T cells. TCR profiling revealed oligoclonal expansion and sharing of activated CD8^+^ T cell clonotypes between MPE and blood. Finally, we found that the blood was a more abundant source of T cell phenotypes with prognostic significance, with high expression of PD-1 on circulating CD4^+^ T cells an independent prognostic factor for poor overall survival. It is also important to note that the patient population had not undergone procedures such as surgery, talc pleurodesis and any type of immunotherapy which could potentially alter the immunological composition of any residual MPE.

CD8^+^ and CD4^+^ T cells in the MPE were characterised by co-expression of multiple IhRs. PD-1 was the predominant IhR expressed by over 50% of MPE CD8^+^ and CD4^+^ T cells, similar to previous reports that studied MPE T cells and TILs in mesothelioma [14, 16, 17, 36, 37]. The higher proportion of total Tregs in MPE compared with matched blood may reflect shifts in CD4^+^ T cell composition that are not apparent when subset frequencies are expressed relative to total Tregs. This may also involve populations outside the activated/resting classification, shaped by the unique cytokine and growth factor milieu of the tumour-associated pleural space [2]. Approximately 5% of CD4^+^ and 10% of CD8^+^ T cells in the MPE co-express 3 or 4 inhibitory receptors in this study. This finding is similar to Prado-Garcia *et al*., where 3–6% of MPE CD8^+^ and CD4^+^ T cells co-expressed PD-1^+^TIM-3^+^ or PD-1^+^LAG-3^+^ in lung cancer patients pre-treatment [38]. Recent single cell analysis of MPE mononuclear cells from 5 lung adenocarcinoma patients also found similar proportions of CD8^+^ T cells with an exhaustion signature that includes co-expression of IhR genes [39]. IhR co-expression on CD8^+^ T cells often indicates diminished effector function, suboptimal T cell activation caused by chronic antigen exposure in a highly immunosuppressive tumour microenvironment [40]. However, CD8^+^ T cells that co-express multiple IhRs still retain some proliferative, cytokine secretion and cytotoxic capacity [41]. Co-expression of IhRs coincided with expression of proliferative (Ki67) and activation (ICOS) markers on subsets of cells in our study. This suggests that MPE T cells could still be responsive to therapies such as ICB. Our observation that the MPE are enriched for memory GzB^+^PD-1^+^ CD8^+^ T cells aligns with studies showing that PD-1^+^ mesothelioma TILs retain cytotoxic function and may be responsive to immunotherapy [16]. This is crucial for the application of intrapleural therapies and harnessing MPE T cells as a potential source for tumour-specific T cell therapy. Surface markers expressed exclusively by tumour-antigen specific, and not bystander TILs have been identified [42]. Although no individual T cell surface marker is an accurate predictor of tumour-antigen specificity, multiple studies suggest that expression of multiple IhRs such as PD-1, LAG-3, TIM-3, the integrin protein CD103 and oligoclonality of CD8^+^ T cells are associated with tumour-specificity [40]. Our study likewise reports these markers on MPE T cells, supporting existing data that some MPE T cells could be expanding due to local antigen-presentation [43]. We identify T cell subsets that can be further interrogated for tumour-antigen specificity.

Co-expression of IhRs on CD4^+^ T cells have been reported in other clinical studies [43, 44], but the expression in relation to CD4^+^ T cell function and differentiation is not well understood. CD4^+^ T cells in the MPE displayed co-expression of IhRs in a similar fashion to CD4^+^ TILs in lung cancer [44–48]. High levels of CD4^+^ Tregs were also likewise previously found in MPEs [49–51].

We compared TCRβ clonotype distribution and overlap between MPE and blood T cells. Both compartments displayed oligoclonal expansion of TCRβ clonotypes, with CD8^+^ clonotypes more likely to traffic between MPE and blood. Oligoclonal expansion of CD8^+^ TCRβ clonotypes in peri-tumoural fluid (ascites or effusion) and matched blood samples were observed in ovarian cancer and lung cancer patients [39, 52, 53]. Oligoclonal expansion of T cells in the blood could be occurring because of systemic infections, or restricted TCRβ repertoires associated with ageing populations in our study [54]. CD8^+^ T cells from matched MPE and blood shared the most TCRβ clones in our study, suggesting trafficking of CD8^+^ T cells between the two compartments. Recent studies highlight the importance of understanding the phenotypes of shared T cell clones between blood and tumours, as shared T cell clones in the blood are phenotypically distinct from their tumour counterparts [55, 56]. These studies suggest that TILs that migrate from the tumour to the circulation lose their exhaustion signature, can be monitored based on surface marker expression, and interrogated for tumour-antigen specificity. Furthermore, increased overlap of TCRβ clones between blood and tumours associated with clinical response to anti-PD-1 ICB [57]. MPE CD8^+^ T cells shared TCRβ clones with blood in a similar fashion to TILs reported in the literature, and we found enrichment of the activated and exhausted T cells in the MPE but not in the blood. It is likely that MPE T cells follow the same pattern as TILs and therefore monitoring shared clones between MPE and blood will greatly help our understanding of mesothelioma-specific T cell responses, especially when tumour biopsies are not available.

Higher proportions of memory T cells correlate with positive clinical outcomes in multiple cancer types [58, 59]. We found higher proportions of CD8^+^ and CD4^+^ T_CM_ enriched in MPE compared to blood, similar to other studies [60–62]. CD8^+^ and CD4^+^ T_RM_ are unique to MPE, similar to previous reports of T_RM_ in ascites and pleural fluid from patients with various malignant tumours [63]. The high levels of TGFβ reported in the MPE could be driving the upregulation of CD103 and CD69, leading to the maintenance and retention of T_RM_ in the MPE [20, 64]. Similar to other reports, intratumoural T_RM_ frequently express multiple IhRs [65, 66] and have clonally expanded TCRβ repertoires [67, 68]. Despite the prognostic significance of these cells when present within the tumour microenvironment [69], their abundance within MPE was not predictive of survival in this patient cohort. Given that T_RM_ have been identified as tumour reactive T cells [70] and linked with enhanced response to immunotherapy [71–73], further investigation of this subset within MPE is warranted as a source of mesothelioma specific T cells, a potential biomarker predictive of response to immunotherapy or a therapeutic target.

Despite the presence of unique T cell subsets within the MPE and enriched expression of IhRs, the blood was the source of more robust T cell biomarkers prognostic for survival. Several paired subsets were prognostic upon univariate analysis, including the proportions of CD8^+^ T_EMRA_ and CD4^+^PD-1^+^TIGIT^+^ T cells in both the MPE and blood. Despite enriched expression of IhR on CD8^+^ T cells in MPE, their abundance was not linked with survival in this patient cohort. Various lung cancer studies report absolute numbers of MPE T cells predict overall survival, including in the context of ICB [74–76]. Huang and colleagues defined a MPE specific T cell exhaustion gene signature that could be mapped to tumour samples and predict poor prognosis in lung cancer patients [39]. Stem like memory T cells in MPE correlated with improved overall survival in lung cancer and mesothelioma [37]. In contrast, Salarolgio and colleagues reported that increased frequencies of CD4^+^PD-1^+^, CD4^+^TIM-3^+^, CD4^+^LAG-3^+^ T cells within tumour tissue, but not MPE correlated with poor survival in mesothelioma patients [14]. Nonetheless, we show that T cell immunity can be tracked in the MPE and therefore potential MPE T cell biomarkers for predicting therapeutic responses requires further investigation.

Here we show that high expression of PD-1 with or without TIGIT on circulating CD4^+^ T cells was an independent predictor of poor survival in this patient cohort. This association between the status of systemic CD4^+^ T cells and survival is consistent with the requirement of a functional CD4^+^ T cell response for the development of effective anti-tumour immunity [77]. This crucial involvement of CD4^+^ T cells in initiating and sustaining an anti-tumour immune response supports the idea that they have potential to be a good predictor of outcome and/or indicator of response to immunotherapy. In line with this, high expression of PD-1 on circulating CD4^+^ T cells was associated with shorter progression-free and overall survival in patients with advanced NSCLC [78, 79] and identified patients who responded poorly to anti-PD-L1 therapy [79]. Similarly, baseline circulating CD4^+^ T cells subsets have been shown to predict the clinical response to PD-1/PD-L1 blockade [78]. Although PD-1^+^ T cells were prevalent in the MPE, response rates to anti-PD1 monotherapy for mesothelioma are reported to be low. Most patients in this cohort did not receive anti-PD-1, which precluded direct assessment of the relationship between PD-1^+^ T cells and clinical response to anti-PD-1 treatment. However, our finding that PD-1 expression on circulating CD4^+^ T cells is prognostic for poor survival in patients with mesothelioma warrants validation in a larger cohort of patients and further investigation as a biomarker of response to immunotherapy in this patient group.

There are several limitations to this study. Firstly, analysis was performed on samples collected from a single time-point, whereas dynamic T cell changes, particularly over the first few cycles of treatment could offer greater insight into delineating responders from non-responders to therapy [80]. Secondly, there is a lack of in-depth T cell functional analyses, such as cytokine production in response to stimuli [81], assessment of chromatin accessibility along key transcription regulators such as TOX [82]. Lastly, the MPE and tumour microenvironment are complex, and contributions of other immune cells such as B cells, NK cells, myeloid derived suppressor cells, macrophages, along with their variable expression of PD-1/L1 could influence biomarker development and therapeutic outcomes.

In conclusion, in-depth characterisation of MPE T cells demonstrates that the MPE provides a unique source to track the anti-tumour T cell response for cancer patients where tumour biopsies are difficult to obtain. This study provides the basis to investigate the use of this compartment for longitudinal monitoring of anti-tumour T cell response in further work however, it would rely on the ongoing presence of a pleural effusion. This study lays the groundwork for future research to identify MPE based T cell biomarkers to predict treatment outcomes particularly for cancer immunotherapies.

## Supplementary Material

iqaf008_Supplementary_Data

## Data Availability

The data underlying this article will be shared on reasonable request to the corresponding author.
