# Supplementary material for: Comparative analysis of malignant pleural effusion and peripheral blood reveals unique T cell signatures associated with survival in mesothelioma patients
Source: Oxf Open Immunol. 2025 Dec 24;7(1):iqaf008. doi: 10.1093/oxfimm/iqaf008 (PMC12823008; doi:10.1093/oxfimm/iqaf008)
Supplement: iqaf008_Supplementary_Data [file iqaf008_supplementary_data.zip › Supplementary Data.docx]

**SUPPLEMENTARY DATA**

***Comparative analysis of malignant pleural effusion and peripheral blood reveals unique T cell signatures associated with survival in mesothelioma patients***

***Principe et al.,***

**Table S1.** **Flow cytometry antibodies for T cell phenotyping**

| **Fluorochrome** | **Antigen** | **Dilution** | **Clone** | **Isotype** | **Supplier** | **Catalog No.** | **Panel** |
| --- | --- | --- | --- | --- | --- | --- | --- |
| BUV395 | CD45 | 1:200 | HI30 | mIgG1 | BD | 563792 | 1,2 |
| BV510 | CD3 | 1:100 | UCHT1 | mIgG1 | BD | 563109 | 1,2 |
| BV711 | CD4 | 1:200 | OKT4 | mIgG2b | BioLegend | 317440 | 1,2 |
| BV785 | CD45RA | 1:100 | HI100 | mIgG2b | BioLegend | 304140 | 1,2 |
| PE-Cy7 | PD-1 | 1:50 | EH12.2H7 | mIgG1 | BioLegend | 329918 | 1,2 |
| eFluor 780 | Fixable Viability | 1:4000 | n/a | n/a | eBioscience | 65-0865-14 | 1,2 |
| BUV737 | CD127 | 1:50 | HIL-7R-M21 | mIgG1 | BD | 564300 | 1 |
| BV421 | CD25 | 1:50 | M-A251 | mIgG1 | BD | 562442 | 1 |
| eVolve 655 | CD8 | 1:300 | RPA-T8 | mIgG1 | eBioscience | 86-0088-41 (25T) | 1 |
| FITC | Ki67 | 1:20 | B56 | mIgG1 | BD | 556026 | 1 |
| PerCP-eF710 | ICOS | 1:50 | ISA-3 | mIgG1 | eBioscience | 46-9948-42 | 1 |
| PE | Foxp3 | 1:40 | 236A/E7 | mIgG1 | eBioscience | 12-4777-42 | 1 |
| PE-CF594 | OX40 | 1:20 | ACT35 | mIgG1 | BD | 563662 | 1 |
| APC | TIGIT | 1:50 | MBSA43 | mIgG1 | eBioscience | 17-9500-42 | 1 |
| BUV737 | CD95 | 1:200 | DX2 | mIgG1 | BD | 564710 | 2 |
| BV421 | CD103 | 1:100 | Ber-ACT8 | mIgG1 | BioLegend | 350214 | 2 |
| BV605 | Tim-3 | 1:40 | F38-2E2 | mIgG1 | BioLegend | 345018 | 2 |
| AF488 | CCR7 | 1:20 | G043H7 | mIgG2a | BioLegend | 353206 | 2 |
| PerCP-eF710 | CD8a | 1:400 | SK1 | mIgG1 | eBioscience | 46-0087-42 | 2 |
| PE | LAG-3 | 1:50 | 3DS223H | mIgG1 | eBioscience | 12-2239-42 | 2 |
| PE-CF594 | CD69 | 1:100 | FN50 | mIgG1 | BD | 562617 | 2 |
| AF647 | TIGIT | 1:50 | MBSA43 | mIgG1 | eBioscience | 17-9500-42 | 2 |
| FITC | CD8 | 1:200 | RPA-T8 | mIgG1 | BioLegend | 301006 | 3 |
| PerCP-Cy5.5 | PD-1 | 1:50 | EH12.2H7 | mIgG1 | BioLegend | 329913 | 3 |
| PE-Cy7 | CCR7 | 1:50 | G043H7 | mIgG2a | BioLegend | 353225 | 3 |
| APC-eF780 | CD14 | 1:100 | 61D3 | mIgG1 | eBioscience | 47-0149-42 | 1,2,3 |
| APC-eF780 | CD19 | 1:100 | HIB19 | mIgG1 | eBioscience | 47-0199-42 | 1,2,3 |

**Table S2**. **Flow cytometry antibodies for T cell sorting.**

| **Fluorochrome** | **Antigen** | **Dilution** | **Clone** | **Isotype** | **Supplier** | **Catalog No.** |
| --- | --- | --- | --- | --- | --- | --- |
| BV510 | CD3 | 1:100 | UCHT1 | mIgG1 | BD | 563109 |
| BV650 | CD8 | 1:500 | SK1 | mIgG1 | BD | 565289 |
| BV711 | CD4 | 1:200 | OKT4 | mIgG2b | BioLegend | 317440 |
| PE | PD-1 | 1:50 | EH12.2H7 | mIgG1 | BioLegend | 329905 |
| FITC | CD45 | 1:50 | HI30 | mIgG1 | BD | 55482 |
| eFluor 780 | Fixable Viability | 1:4000 | n/a | n/a | eBioscience | 65-0865-14 |

**Table S3. Univariate cox regression analysis of immunological variables.**

|  | **HR** | **95% CI** | **P-value** |
| --- | --- | --- | --- |
| ***Peripheral Blood Variables*** |  |  |  |
| % Ki67^+^ CD4^+^ Treg cells  % CD4^+^ T_N_ cells  % CD4^+^ T_CM_ cells  % CD4^+^ T_EM_ cells  % CD8^+^ T_CM_ cells  % CD8^+^ T_EM_ cells  % CD8^+^ TIGIT^+^ T cells  % CD8^+^ PD-1^+^TIGIT^+^ T cells | 1.01  0.98  1.03  1.02  0.96  1.00  1.00  0.97 | 0.95 - 1.07  0.95 - 1.00  1.00 - 1.06  0.95 - 1.08  0.89 - 1.02  0.96 - 1.04  0.97 - 1.03  0.92 - 1.01 | 0.821  0.084  0.060  0.626  0.206  0.907  0.911  0.177 |
|  |  |  |  |
| ***MPE Variables*** |  |  |  |
| % Ki67^+^ CD4^+^ Treg cells  % CD4^+^ T_N_ cells  % CD4^+^ T_CM_ cells  % CD4^+^ T_EM_ cells  % CD4^+^ T_RM1_ cells  % CD4^+^ T_RM2_ cells  % CD4^+^ TIM-3^+^ T cells  % CD4^+^ LAG-3^+^ T cells  % CD8^+^ T_CM_ cells  % CD8^+^ T_EM_ cells  % CD8^+^ TIGIT^+^ T cells  % CD8^+^ TIM-3^+^ T cells  % CD8^+^ LAG-3^+^ T cells  % CD8^+^ PD-1^+^TIGIT^+^ T cells | 1.01  0.97  1.00  1.01  1.01  1.04  1.04  1.02  1.00  1.00  1.01  1.04  0.98  1.00 | 0.98 – 1.05  0.94 – 1.01  0.98 – 1.03  0.99 – 1.03  0.98 – 1.04  0.99 – 1.09  0.89 – 1.21  0.84 – 1.23  0.98 - 1.03  0.97 – 1.03  0.98 – 1.04  0.98 – 1.10  0.92 – 1.05  0.97 – 1.02 | 0.459  0.122  0.719  0.279  0.645  0.089  0.651  0.875  0.918  0.956  0.430  0.200  0.626  0.808 |

*HR* hazard ratio, *CI* confidence interval, *MPE* malignant pleural effusion, *T_N_* naïve T cells (CD45RA^+^CCR7^+^), *T_CM_* central memory T cells (CD45RA^-^CCR7^+^), *T_EM_* effector memory T cells (CD45RA^-^CCR7^-^), *T_RM1_* tissue resident memory T cells subset 1 (CD69^+^CD103^-^), *T_RM2_* tissue resident memory T cells subset 2 (CD69^+^CD103^+^).

**Table S4. Multivariate cox regression backward stepwise elimination summary**

| **Step** | **Variable** | **R^2^** | **Adj R^2^** | **C(p)** | **RMSE** |
| --- | --- | --- | --- | --- | --- |
|  | ***Model 1*** |  |  |  |  |
| 1  2  3  4  5  6 | MPE % CD4^+^ PD-1^+^TIGIT^+^ T cells  MPE % CD4^+^ Ki67^+^ T cells  PB % CD4^+^ TIGIT^+^ T cells  MPE % CD8^+^ T_EMRA_ cells  PB % CD8^+^ T_N_ cells  PB % CD8^+^ T_EMRA_ cells | 0.590  0.588  0.581  0.572  0.566  0.537 | 0.433  0.456  0.473  0.483  0.496  0.484 | 8.182  6.288  4.567  3.034  1.369  0.761 | 10.556  10.340  10.183  10.081  9.955  10.075 |
|  |  |  |  |  |  |
|  | ***Model 2*** |  |  |  |  |
| 1  2  3  4  5 | MPE % CD4^+^ PD-1^+^TIGIT^+^ T cells  PB % CD8^+^ T_N_ cells  MPE % CD4^+^ Ki67^+^ T cells  MPE % CD8^+^ T_EMRA_ cells  PB % CD8^+^ T_EMRA_ cells | 0.611  0.604  0.594  0.582  0.545 | 0.487  0.501  0.510  0.515  0.492 | 7.091  5.450  3.975  2.662  2.664 | 10.047  9.910  9.819  9.770  9.994 |

MPE malignant pleural effusion, PB peripheral blood, TN naïve T cells, Adj adusted, C(p) Mallows’ Cp, RMSE root mean square error.

**
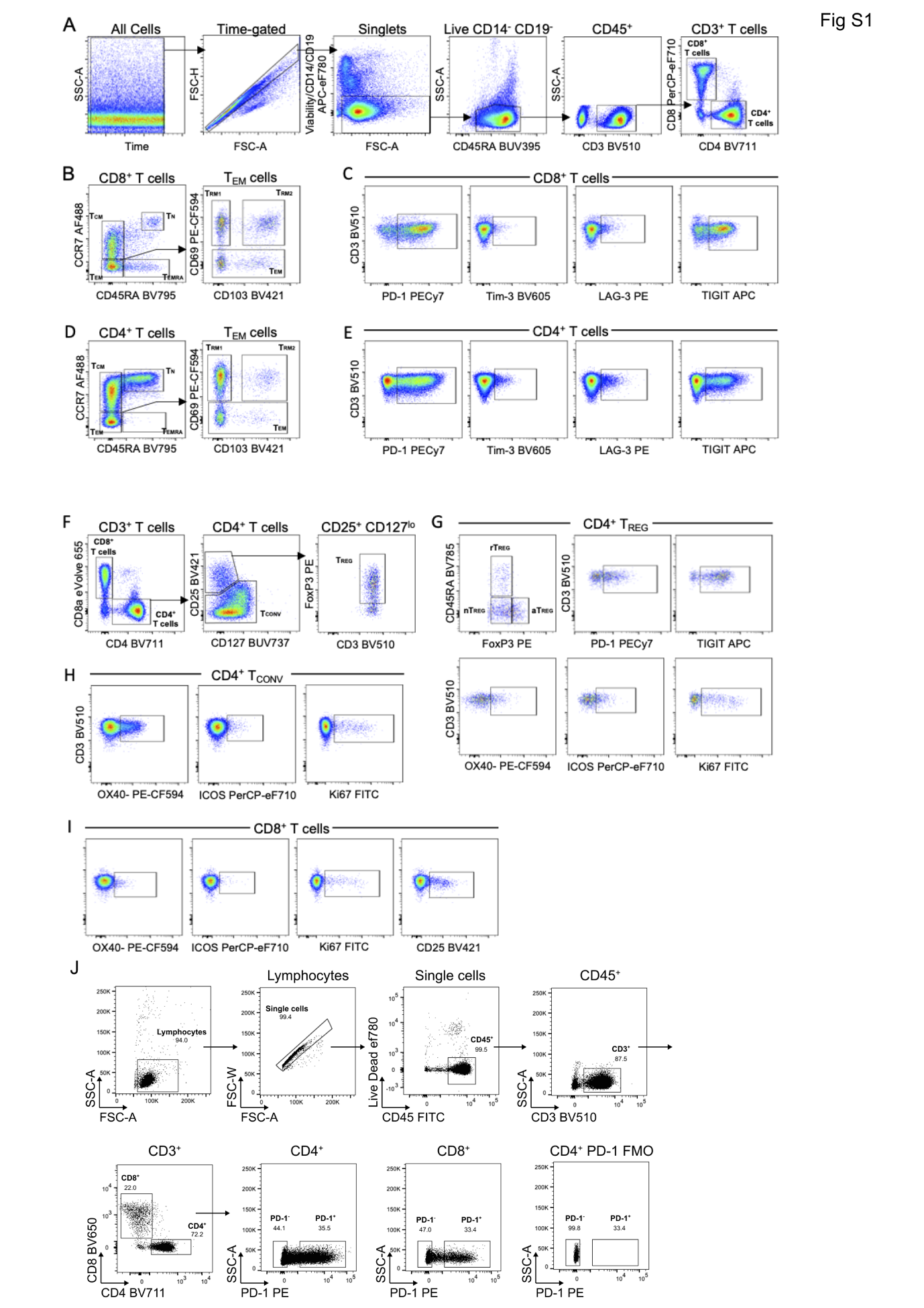
**

**Figure S1. Flow cytometry gating strategy for T cell phenotyping and T cell sorting. A)** Parent gating for both T cell phenotyping panels. **B-E)** Gating for memory subsets and inhibitory receptor expression for CD8^+^ and CD4^+^ T cells. **F)** Gates for regulatory T cell subsets (Treg). **G-I)** Gates for activation, stimulation, and inhibitory receptors for Treg**,** Tconv and CD8^+^ T cells. **J)** Gating strategy for sorting CD8^+^PD-1^+^ and CD4^+^PD-1^+^ T cells.


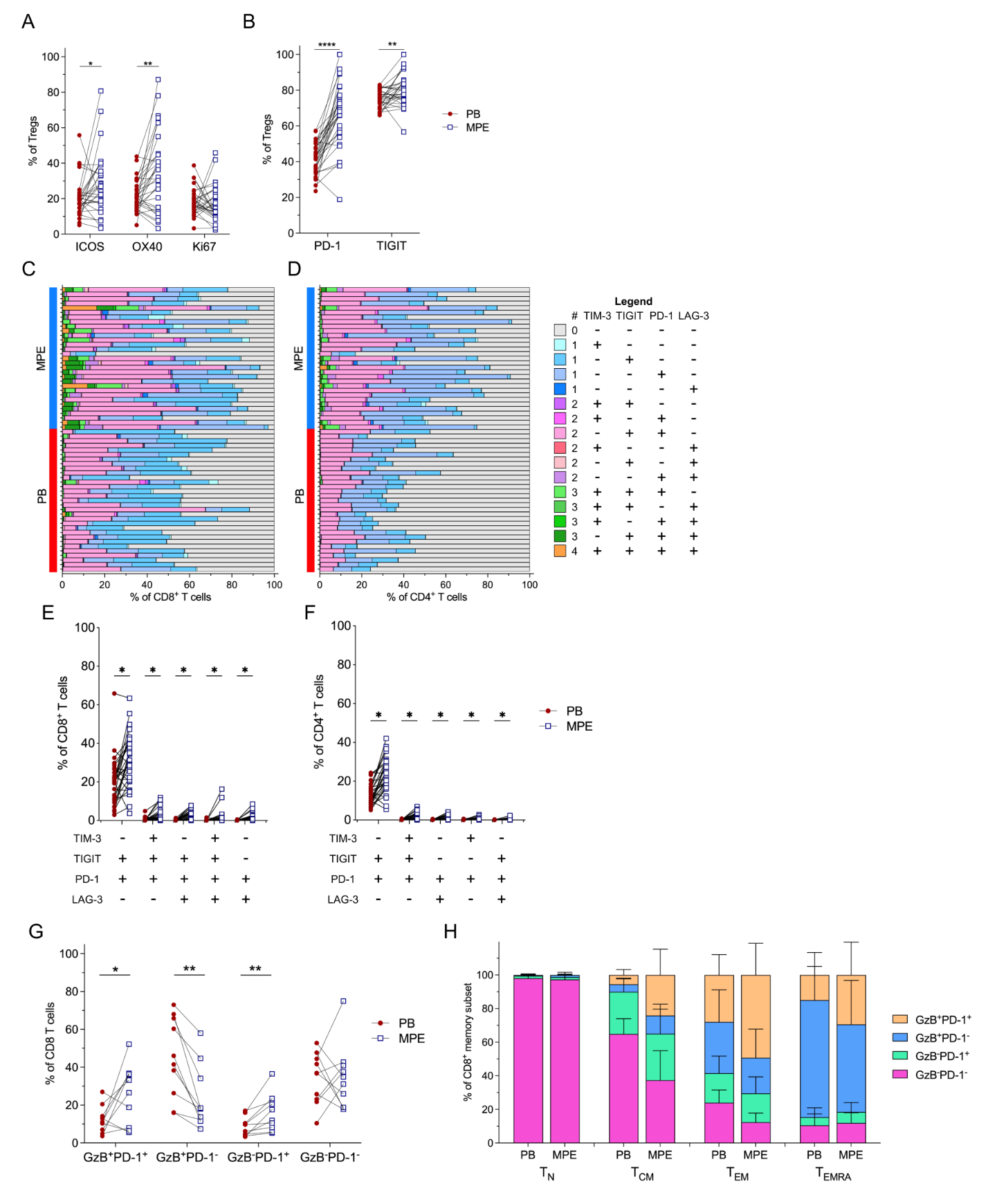


**Figure S2. MPE T cells exhibit increased inhibitory receptor expression. A)** Frequency of activation markers; ICOS, OX40 and Ki67 expressed on Tregs in the PB and MPE. **B**) Proportion of inhibitory receptors (IhR): PD-1 and TIGIT on Tregs in the PB and MPE**.** Lines connecting PB and MPE samples represent samples obtained from the same patient. **C-D)** Stacked bar plots representing the proportion of CD8^+^ and CD4^+^ T cells co-expressing PD-1, TIGIT, TIM-3 and LAG-3, coloured by T cells expressing only 1 IhR, any 2 IhRs, any 3 IhRs or all 4 IhRs. Each bar represents a PB or MPE sample from each patient. **E-F)** Frequency of the five most abundant co-expressed IhR combinations (2 or more IhRs) in CD8^+^ **(E)** and CD4^+^ **(F)** T cells. **G)** Frequency of CD8^+^ T cells co-expressing PD-1 and granzyme B (GzB) in PB and MPE. **H)** Stacked bar plots showing PD-1 and GzB expression on CD8^+^ memory T cell subsets in PB and MPE. Paired t-test was used for normally distributed data, otherwise Wilcoxon matched-pairs signed-rank test was used. *P<0.05, **P<0.01, ***P<0.001, ****P<0.0001

**
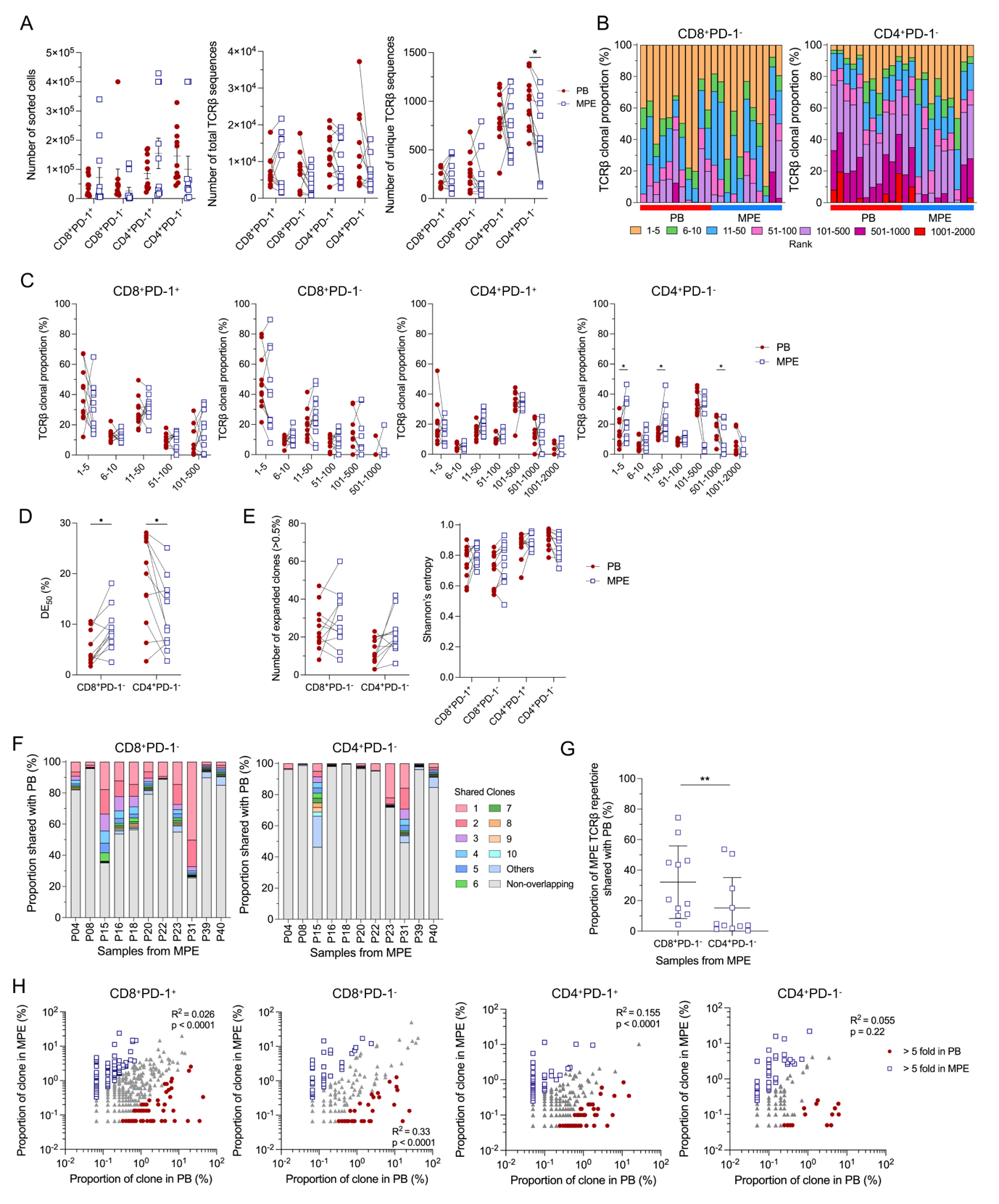
**

**Figure S3. PB TCRβ repertoires shares clones found in the MPE. A)** Number of sorted cells, total and unique TCRβ sequences collected from bulk TCRβseq of CD8^+^PD-1^+^, CD8^+^PD-1^-^, CD4^+^PD-1^+^ CD4^+^PD-1^-^ T cells from paired PB and MPE. **B)** Distribution of TCRβ clones, ranked by total abundance in CD8^+^PD-1^-^ (left) and CD4^+^PD-1^-^ (right) TCRβ repertoires. Each bar represents the repertoire for each patient sample. **C)** Percentage of TCRβ clones, grouped in ranked order of most abundant to least abundant clone between paired PB and MPE for each TCRβ repertoires. **D)** Diversity evenness (DE_50_) between paired PB and MPE for CD8^+^PD-1^-^ and CD4^+^PD-1^-^ TCRβ repertoires. **E)** Number of expanded clones (left) and Shannon’s entropy (right) for each TCRβ repertoire. Lower Shannon’s entropy indicates a less diverse, more clonal repertoire. **F)** Bar plots of MPE CD8^+^PD-1^-^ (left) and CD4^+^PD-1^-^ (right) TCRβ repertoires, coloured by proportion of the most abundant TCRβ clones shared with paired PB. Each bar represents MPE TCRβ repertoire for each patient. **G)** Proportion of clones in MPE CD8^+^PD-1^-^ and CD4^+^PD‑1^-^ TCRβ repertoires also found in paired PB. **H)** Dot plot representing the combined distribution of shared clones in the PB and MPE for CD8^+^PD-1^+^, CD8^+^PD-1^-^, CD4^+^PD-1^+^ and CD4^+^PD-1^-^ TCRβ repertoires. Shared clones are coloured if the frequency is 5-fold greater in the PB (right) or MPE (blue). Lines connecting PB and MPE samples represent samples obtained from the same patient. Data presented as median[IQR] in A, G, I. Paired t-test was used for normally distributed data, otherwise Wilcoxon matched-pairs signed-rank test was used. *P<0.05, **P<0.01, ***P<0.001, ****P<0.0001.


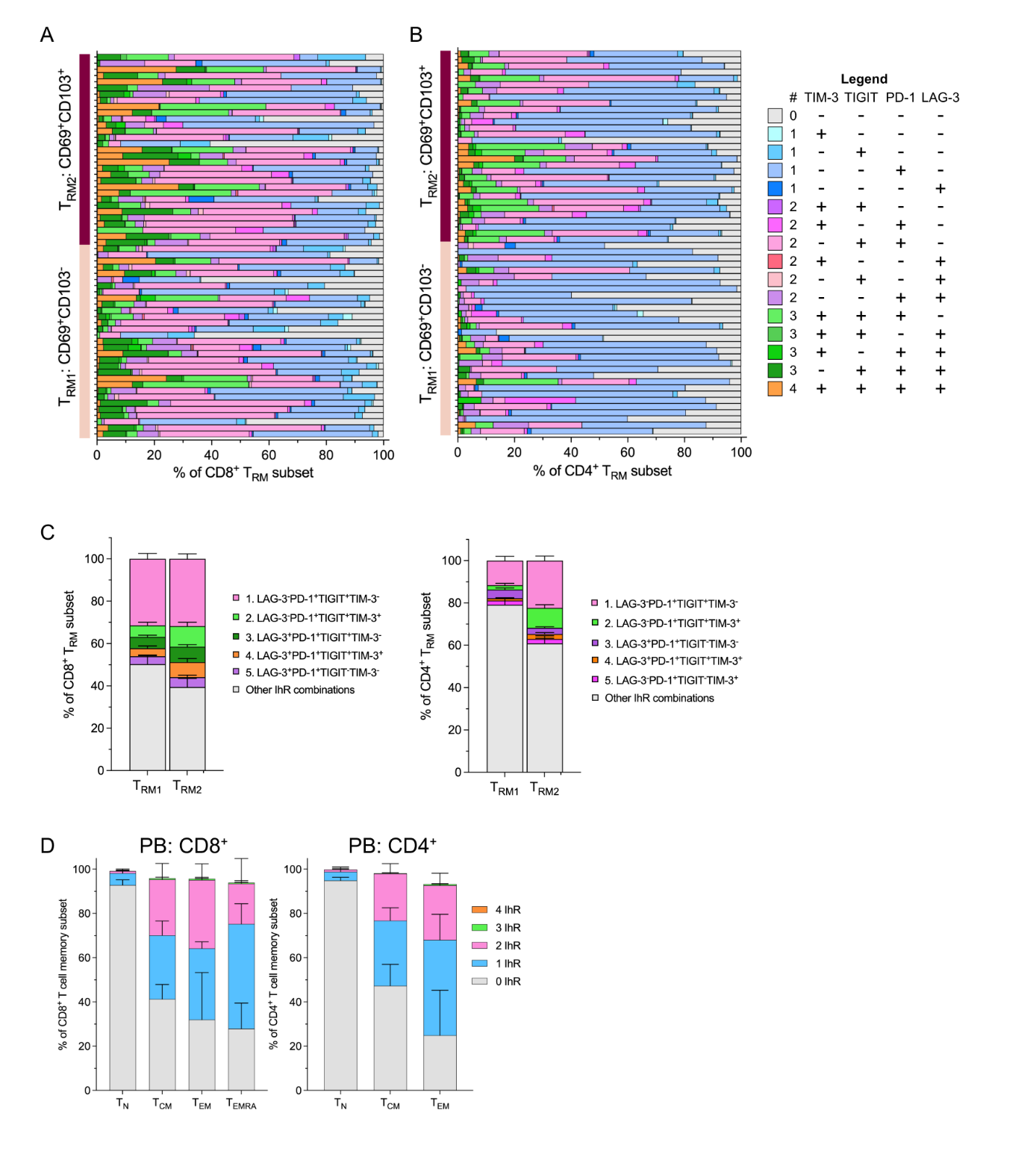


**Figure S4. T_RMs_ in the MPE co-express inhibitory receptors. A-B)** Stacked bar plots representing the proportion of CD8^+^ (left) and CD4^+^ (right) T_RM1_ and T_RM2_ cells co-expressing PD-1, TIGIT, TIM-3 and LAG-3, coloured by T cells expressing only 1 IhR, any 2 IhRs, any 3 IhRs or all 4 IhRs. Each bar represents a MPE sample from each patient. **C)** Stacked bar plots showing the five most abundant co-expressed IhR combinations (2 or more IhRs) in CD8^+^ (left) and CD4^+^ (right) T_RM1_ and T_RM2_ cells. **D)** Stacked bar plot showing IhR expression on memory subsets in PB CD8^+^ (left) and CD4^+^ (right) T cells. The number of PB CD4^+^ T_EMRA_ cells was too low for accurate phenotypic analysis. Data presented as mean±SEM in C, median [IQR] in D.

**
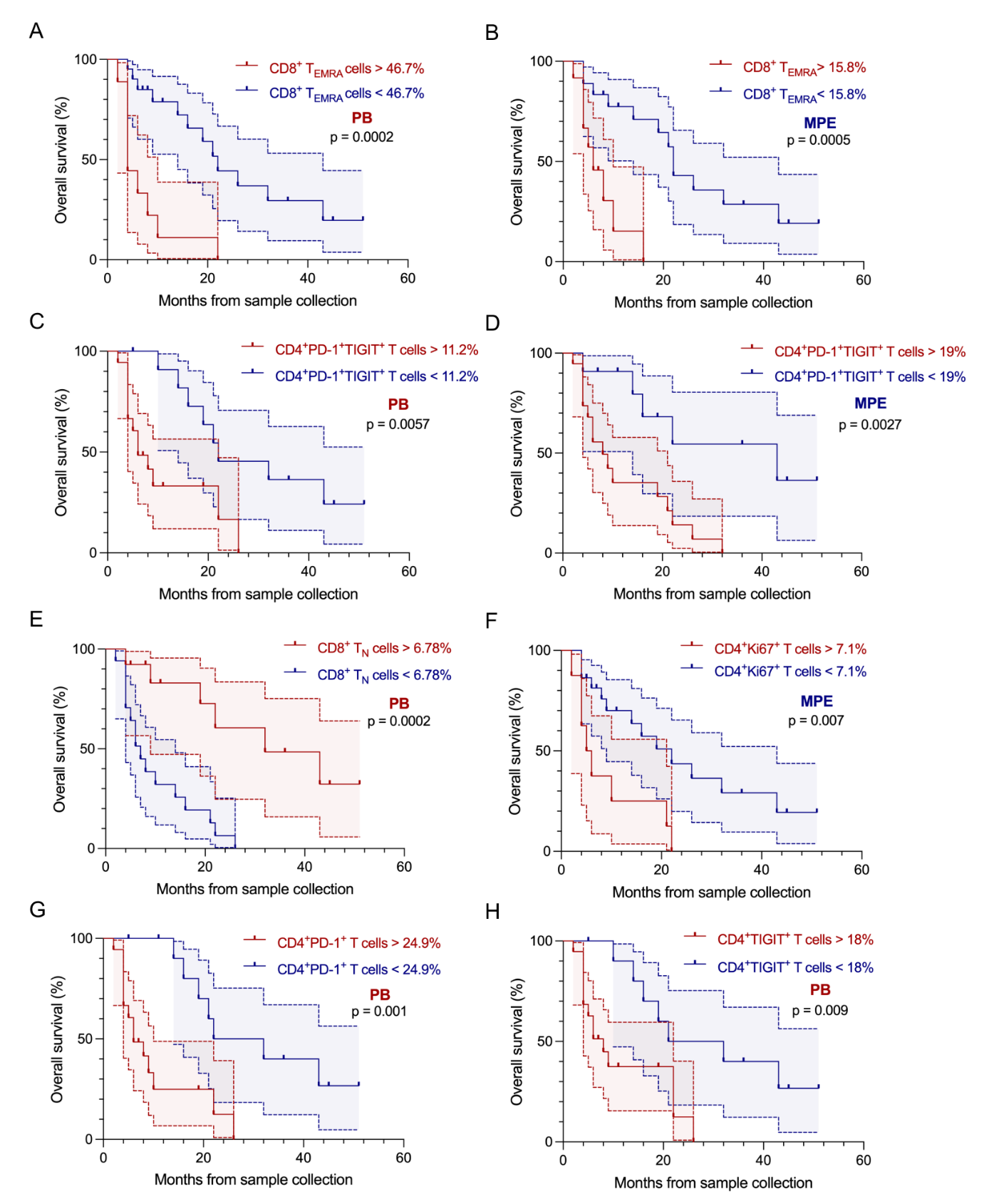
**

**Figure S5. MPE and blood T cell subsets predict survival in mesothelioma patients.** Kaplain-Meir plots of 31 mesothelioma patients separated into two groups based on frequencies of **A)** PB CD8^+^ T_EMRA_, **B)** MPE CD8^+^ T_EMRA_, **C)** PB CD4^+^PD-1^+^TIGIT^+^**, D)** MPE CD4^+^PD-1^+^TIGIT^+^**,**  **E )**PB CD8^+^ T_N_ , **F)** MPE CD4^+^Ki67^+^**, G)** PB CD4^+^PD-1^+^ and **H)** PB CD4^+^TIGIT^+^ T cells**.** Data were dichotomized at the point demonstrating the strongest association with survival in univariate regression analysis (26).


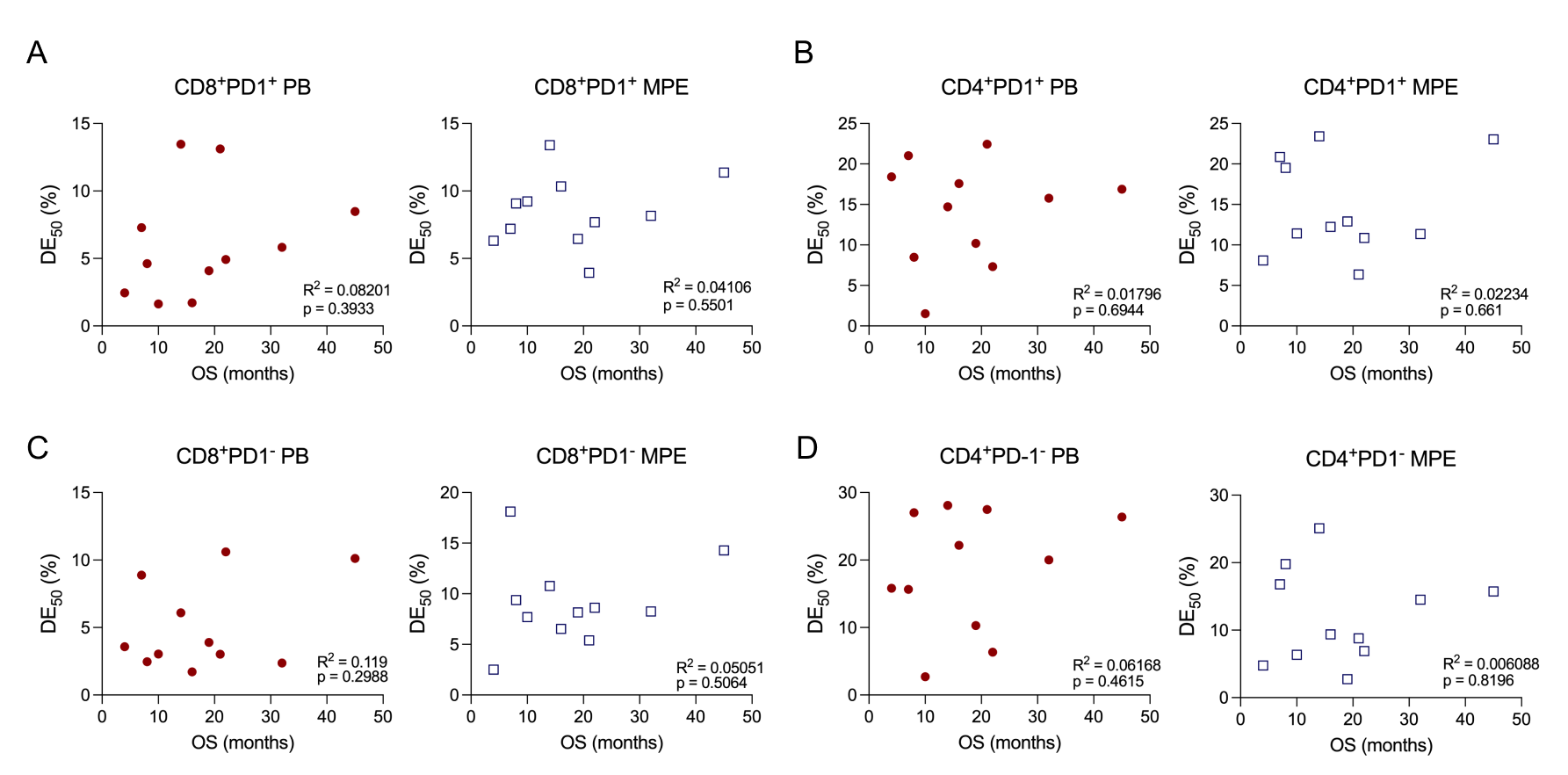


**Figure S6. TCRβ repertoire clonality does not correlate with survival in mesothelioma patients.** Correlation plots of overall survival with DE50 of CD8^+^PD-1^+^ (A), CD4^+^PD-1^+^ (B), CD8^+^PD-1^-^ (C) and CD4^+^PD-1^-^ PB (left) and MPE (right) TCRβ repertoires.
